# Stereotactic vs Hypofractionated Radiotherapy for Inoperable Stage I Non–Small Cell Lung Cancer: The LUSTRE Phase 3 Randomized Clinical Trial

**DOI:** 10.1001/jamaoncol.2024.3089

**Published:** 2024-09-19

**Authors:** Anand Swaminath, Sameer Parpia, Marcin Wierzbicki, Vijayananda Kundapur, Sergio Faria, Gordon S. Okawara, Theodoros K. Tsakiridis, Naseer Ahmed, Alexis Bujold, Khalid Hirmiz, Timothy Owen, Nelson Leong, Kevin Ramchandar, Edith Filion, Harold Lau, Zsolt Gabos, Robert Thompson, Brian Yaremko, Selma Mehiri, Alexander V. Louie, Kimmen Quan, Mark N. Levine, James R. Wright, Timothy J. Whelan

**Affiliations:** 1 Department of Oncology, McMaster University, and the Division of Radiation Oncology Juravinski Cancer Centre at Hamilton Health Sciences, Hamilton, Ontario, Canada; 2 Department of Oncology, McMaster University, Hamilton, Ontario, Canada; 3 Juravinski Cancer Centre, Department of Medical Physics, McMaster University, Hamilton, Ontario, Canada; 4 Saskatchewan Cancer Agency, Department of Radiation Oncology, University of Saskatchewan, Saskatoon, Saskatchewan, Canada; 5 Department of Radiation Oncology, McGill University Health Centre, Montreal, Quebec, Canada; 6 Section of Radiation Oncology, Department of Radiology, Rady Faculty of Health Sciences, University of Manitoba and CancerCare Manitoba Research Institute, Winnipeg, Manitoba, Canada; 7 Département de Radio-oncologie Clinique-Enseignement-Recherche, Centre intégré universitaire de soins et services sociaux de l’Est-de-l’Île-de-Montréal - Hôpital Maisonneuve-Rosemont, Montreal, Quebec, Canada; 8 Department of Radiation Oncology, Windsor Regional Cancer Centre, Windsor, Ontario, Canada; 9 Department of Oncology, Queen’s University, Cancer Centre of Southeast Ontario at Kingston Health Sciences Centre, Kingston, Ontario, Canada; 10 Allan Blair Cancer Centre, Department of Radiation Oncology, University of Saskatchewan, Regina, Saskatchewan, Canada; 11 Department of Oncology, Northern Ontario School of Medicine, Thunder Bay, Ontario, Canada; 12 Radiation Oncology Department, Centre Hospitalier de l’Université de Montréal, Notre Dame Hospital, Montreal, Quebec, Canada; 13 Department of Oncology, University of Calgary, Calgary, Alberta, Canada; 14 Department of Oncology, University of Alberta, Edmonton, Alberta, Canada; 15 Department of Radiation Oncology, Dalhousie University, Saint John, New Brunswick, Canada; 16 Department of Radiation Oncology, Western University, London, Ontario, Canada; 17 Département de Radio-oncologie, CISSS Montérégie, Hôpital Charles Lemoyne, Montreal, Quebec, Canada; 18 Department of Radiation Oncology, University of Toronto, Toronto, Ontario, Canada

## Abstract

**Importance:**

Stereotactic body radiotherapy (SBRT) is widely used for stage I medically inoperable non–small cell lung cancer (NSCLC), yet varied results from randomized clinical trials (RCTs) and concerns in treating centrally located tumors persist.

**Objective:**

To examine whether SBRT would improve local control (LC) compared with hypofractionated conventional radiotherapy (CRT).

**Design, Setting, and Participants:**

This phase 3 RCT was conducted in 16 Canadian centers. Patients with medically inoperable stage I (≤5 cm) NSCLC were randomized 2:1 to SBRT of 48 Gy in 4 fractions (peripheral NSCLC) or 60 Gy in 8 fractions (central NSCLC) vs CRT of 60 Gy in 15 fractions. Data were collected from May 2014 to January 2020, and data were analyzed from July 2022 to July 2023.

**Interventions:**

SBRT or CRT.

**Main Outcomes and Measures:**

The primary objective was to determine the effectiveness of SBRT compared with CRT based on LC at 3 years. Secondary outcomes included event-free survival, overall survival, and toxic effects. All radiation plans were subject to real-time/final review. Local failures were centrally adjudicated. The study was designed to detect a 3-year LC improvement of SBRT from 75% to 87.5%. The target sample size was 324 patients.

**Results:**

Of 233 included patients, 119 (51.1%) were male, and the mean (SD) age was 75.4 (7.7) years; the median (IQR) follow-up was 36.1 (26.4-52.8) months. A total of 154 patients received SBRT and 79 received CRT. The 3-year LC was 87.6% (95% CI, 81.9%-93.4%) for SBRT and 81.2% (95% CI, 71.9%-90.5%) for CRT (hazard ratio [HR], 0.61; 95% CI, 0.31-1.20; *P* = .15). The HR was 1.02 (95% CI, 0.72-1.45; *P* = .87) for event-free survival and 1.18 (95% CI, 0.80-1.76; *P* = .40) for overall survival. Minimal acute toxic effects were observed. Among those randomized to SBRT, late grade 3 or 4 toxic effects occurred in 5 of 45 (11%) with central NSCLC and 2 of 109 (1.8%) with peripheral NSCLC; among those randomized to CRT, in 1 of 19 (5%) with central NSCLC and 1 of 60 (2%) with peripheral NSCLC. One patient who received SBRT for an ultracentral lesion (target overlapping proximal bronchus) experienced a possible treatment-related grade 5 event (hemoptysis).

**Conclusions and Relevance:**

This RCT compared lung SBRT with hypofractionated CRT that included central/ultracentral tumors. No difference was detected in LC between groups. Severe toxic effects were limited, including patients with central tumors. The trial provides important prospective data evaluating SBRT; however, further research is necessary to determine if SBRT is more effective than CRT for peripheral and central NSCLC.

**Trial Registration:**

ClinicalTrials.gov Identifier: NCT03924869

## Key Points

**Question:**

Does stereotactic body radiotherapy (SBRT) improve outcomes compared with hypofractionated conventional radiotherapy (CRT) in patients with peripheral and central stage I non–small cell lung cancer without increased toxic effects?

**Findings:**

In this phase 3 randomized clinical trial, 233 patients were allocated 2:1 to receive SBRT of 48 Gy in 4 fractions (peripheral tumors) or 60 Gy in 8 fractions (central tumors) vs CRT of 60 Gy in 15 fractions. Among patients randomized to SBRT, there was a nonsignificant improvement in local control compared with CRT at 3 years, and severe toxic effects were low, including in patients with central disease.

**Meaning:**

In this randomized clinical trial, SBRT resulted in acceptable tumor control compared with CRT with limited toxic effects.

## Teaser

This randomized clinical trial examines whether stereotactic body radiotherapy improves local control compared with hypofractionated conventional radiotherapy.

## Introduction

The traditional standard of care for stage I non–small cell lung cancer (NSCLC) is surgery.^1^ In patients with medically inoperable disease, conventionally fractionated radiotherapy (CRT) has historically been associated with poor local control (LC) and survival.^2^ Results of stereotactic body radiotherapy (SBRT) mainly from nonrandomized or large retrospective studies has demonstrated LC of 85% to 95% and overall survival of 65% 75% at 2 to 3 years.^3,4^ This apparent improvement compared with CRT has led to the routine adoption of SBRT in many jurisdictions. Two randomized clinical trials (RCTs) comparing SBRT with CRT performed to date have shown inconsistent results.^5,6^ These trials did not compare SBRT with hypofractionated CRT, which may have better disease control than traditional CRT, nor include patients with central or ultracentral tumors, where concerns exist about severe toxic effects.^7^

We report the results of the LUSTRE trial comparing SBRT with hypofractionated CRT for peripheral and central stage I NSCLC.

## Methods

We conducted a multi-institutional, unblinded RCT across 16 Canadian centers from May 2014 to January 2020; the trial protocol can be found in Supplement 1, and the trial sites and investigators can be found in eAppendix 1 in Supplement 2. Eligible patients had medically inoperable T1/T2a N0M0 NSCLC diagnosed histologically or with a suspicious nodule on computed tomography with malignant ^18^F fluorodeoxyglucose positron emission tomography avidity, when a biopsy was considered high risk. Other inclusion and exclusion criteria are outlined in Supplement 1. Patients provided written informed consent, and institutional review board approval was obtained for each site. This study followed the Consolidated Standards of Reporting Trials (CONSORT) reporting guidelines.

Eligible and consenting patients were stratified by tumor size (T1 disease, ≤3 cm; T2a disease, >3 to 5 cm), location (peripheral or central [ie, within 1 cm of mediastinum and/or 2 cm of proximal bronchial tree]), and institution. Patients were randomized 2:1 to either SBRT of 48 Gy in 4 alternate-day fractions (peripheral disease) or 60 Gy in 8 daily fractions (central disease) vs CRT of 60 Gy in 15 daily fractions. SBRT and CRT planning and treatment details were described previously^8^ (eAppendix 2 in Supplement 2). All patients were seen at least once during and 2 weeks after treatment and then every 3 to 12 months up to 60 months (with thorax computed tomography) after randomization. A rigorous radiotherapy quality assurance (RTQA) process included planning guide development (eAppendix 2 in Supplement 2), credentialing (SBRT/CRT test cases), and real-time/final plan review.

Our hypothesis was that SBRT would result in superior LC compared with CRT. LC was defined as time from randomization to primary tumor or marginal failure at any time point, with censoring only at last follow-up or death (eAppendix 3 in Supplement 2). Histological or positron emission tomography confirmation of recurrence was recommended. Local failures were adjudicated centrally. Secondary outcomes included event-free survival, overall survival (defined in eAppendix 3 in Supplement 2), and toxic effects using the National Cancer Institute Common Terminology Criteria for Adverse Events version 4.03.

LC with CRT was estimated to be 75% at 3 years, with an expected increase to 87.5% with SBRT. A minimum of 59 events were required to have 85% power to detect this difference (hazard ratio [HR], 0.46; 2-sided α = .05). Assuming 3 years for recruitment and 2 years of follow-up (with adjustment for losses), 324 patients (216 randomized to SBRT and 108 randomized to CRT) were required. All analyses were intent to treat. LC was estimated using the Kaplan-Meier approach and compared with the log-rank test. Treatment effects and corresponding 95% CIs were estimated using the Cox proportional hazards model. Toxic effects were reported descriptively. An independent data safety monitoring committee (DSMC) reviewed trial progress annually.

Stata, version 16 (StataCorp), and SAS, version 9.4 (SAS Institute), were used in statistical analyses. A 2-tailed *P* < .05 was considered statistically significant.

## Results

**Figure 1.**
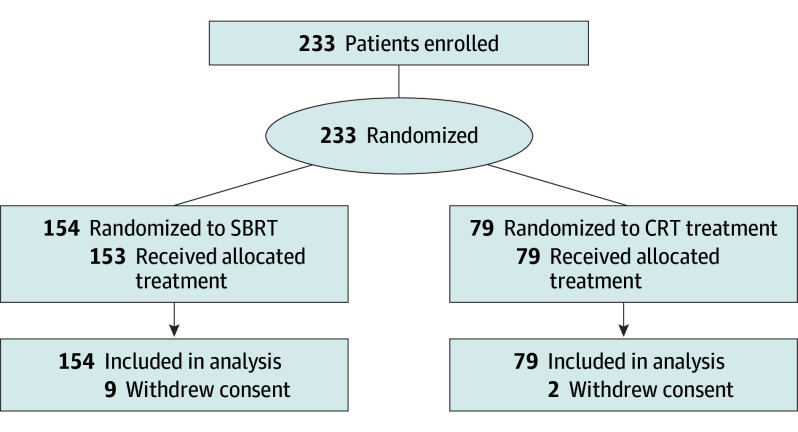
CONSORT Diagram CRT indicates hypofractionated conventional radiotherapy; SBRT, stereotactic body radiotherapy.

A total of 233 patients were randomized (154 to SBRT and 79 to CRT) before the trial closed to slow accrual under DSMC advisement. Of 233 included patients, 119 (51.1%) were male, and the mean (SD) age was 75.4 (7.7) years; the median (IQR) follow-up was 36.1 (26.4-52.8) months. A total of 64 patients (27.5%) had central tumors (45 randomized to SBRT and 19 randomized to CRT). One patient did not receive their intended treatment (SBRT). A total of 9 patients in the SBRT group and 2 in the CRT group withdrew from ongoing follow-up (Figure 1). Baseline characteristics were similar between arms (eAppendix 4 in Supplement 2). Rates of RTQA major and minor deviations were similar (SBRT group: 8 of 154 [5.2%] and 31 of 154 [20.1%], respectively; CRT: 4 of 79 [5.1%] and 11 of 79 [13.9%], respectively).

**Figure 2.**
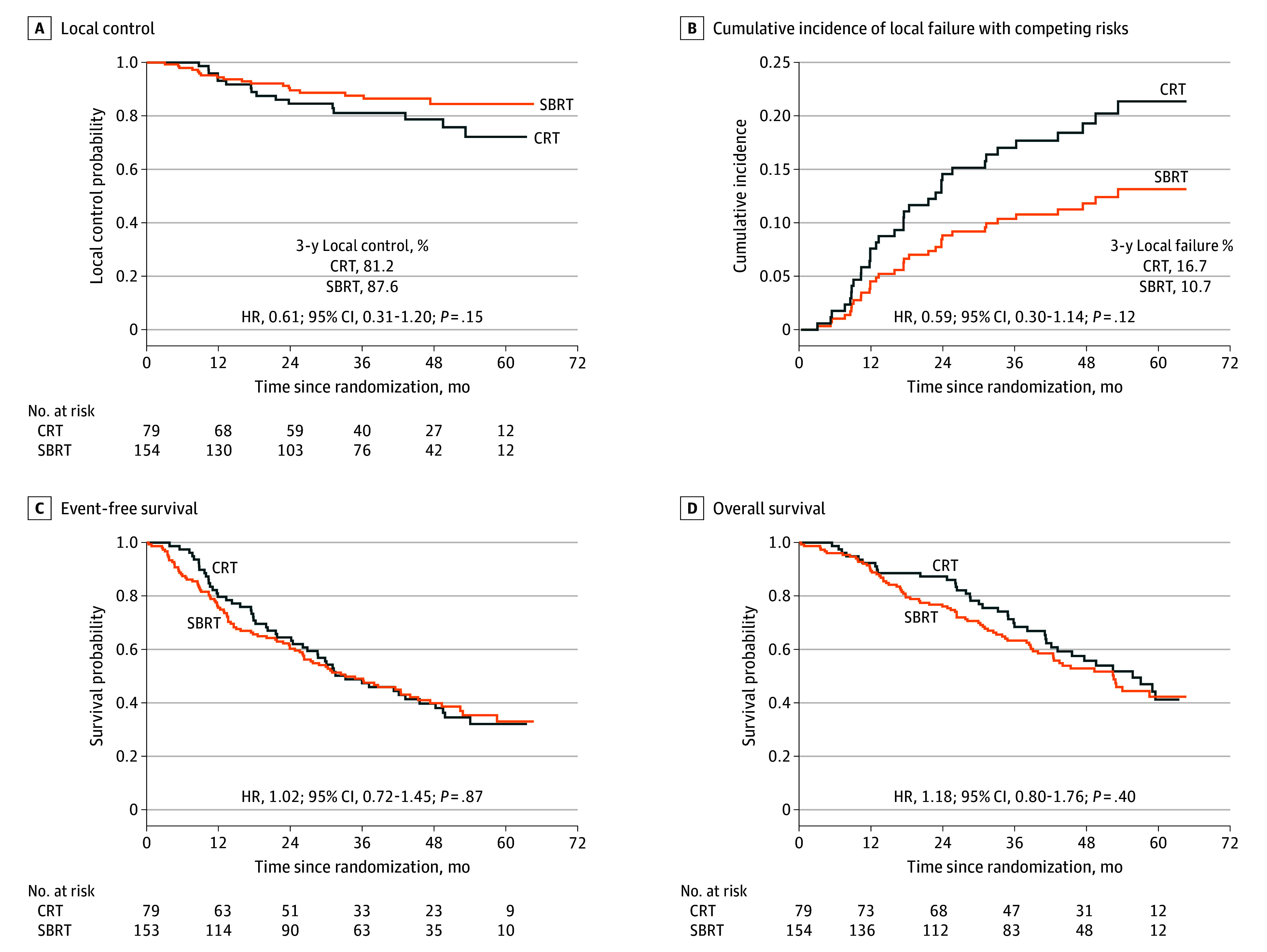
Kaplan-Meier Curves for Outcomes CRT indicates hypofractionated conventional radiotherapy; HR, hazard ratio; SBRT, stereotactic body radiotherapy.

A total of 34 LC events were observed (18 in the SBRT group and 16 in the CRT group). Three-year LC with SBRT was 87.6% (95% CI, 81.9%-93.4%) compared with 81.2% (95% CI, 71.9%-90.5%) for CRT (HR, 0.61; 95% CI, 0.31-1.20; *P* = .15) (Figure 2A). Cumulative incidence of local failure with death as a competing risk yielded similar results (eAppendix 5 in Supplement 2; Figure 2B). Three-year event-free survival was 49.1% (95% CI, 41.0%-57.2%) with SBRT and 47.5% (95% CI, 36.3%-58.6%) with CRT (HR, 1.02; 95% CI, 0.72-1.45; *P* = .87) (Figure 2C), and 3-year overall survival with SBRT was 63.5% (95% CI, 55.6%-71.2%) and 68.4% (95% CI, 58.0%-79.0%) with CRT (HR, 1.18; 95% CI, 0.80-1.76; *P* = .40) (Figure 2D). Patterns of failure and causes of death are summarized in eAppendix 5 in Supplement 2.

One patient in each arm developed grade 3 acute toxic effects (SBRT group: fatigue/pneumonia; CRT group: dyspnea), with no grade 4 or 5 effects observed (Table). A total of 8 (5.2%) in the SBRT group and 2 (3%) in the CRT group developed grade 3 or 4 toxic effects (Table; eAppendix 6 in Supplement 2). For central tumors, grade 3 or higher late toxic effects occurred in 6 of 45 patients (13%) in the SBRT group and 1 of 19 patients (5%) in the CRT group. One grade 5 hemoptysis at 12 months occurred in a patient randomized to SBRT (60 Gy in 8 fractions) with an ultra-central NSCLC (tumor overlapping proximal bronchus). Altogether, 30 patients with ultracentral NSCLC were identified (23 in the SBRT group and 7 in the CRT group), of which 4 in the SBRT group developed grade 3 or higher late toxic effects.

**Table.** Worst Toxic Effects By Grade and Tumor Location

Toxic effect	No. (%)	
	SBRT	CRT
**Worst grade**		
Total, No.	154	79
Acute toxic effects (≤3 mo)		
Grade 1	41 (27)	21 (27)
Grade 2	11 (7)	4 (5)
Grade 3	1 (<1)	1 (1)
Long-term toxic effects (>3-36 mo)		
Grade 1	39 (25)	17 (22)
Grade 2	21 (14)	10 (13)
Grade 3	7 (5)	1 (1)
Grade 4	0	1 (1)
Grade 5	1 (<1)	0
**Grade 3-5 peripheral tumors only**		
Total, No.	109	60
Long-term toxic effects (>3-36 mo)		
Grade 3	2 (2)	1 (2)
Grade 4	0	0
Grade 5	0	0
**Grade 3-5 central tumors only**		
Total, No.	45	19
Long-term toxic effects (>3-36 mo)		
Grade 3	5 (11)	0
Grade 4	0	1 (5)
Grade 5	1 (2)	0

Abbreviations: CRT, hypofractionated conventional radiotherapy; SBRT, stereotactic body radiotherapy.

## Discussion

To our knowledge, the LUSTRE trial is the largest reported NSCLC SBRT RCT to date. SBRT was associated with a nonsignificant improvement in LC compared with hypofractionated CRT. During active accrual, SBRT was increasingly adopted due to convenience. Efforts were made to encourage accrual particularly for central NSCLC, but ultimately the trial closed on a DSMC recommendation just before the COVID-19 pandemic.^9^

SBRT LC rates were similar to other reported trials. CRT hypofractionation was based on the Canadian BR.25 trial^10^ and performed better than expected, which may have contributed to the nonsignificant difference observed between groups. Modest hypofractionation could possibly be used as a low-risk SBRT alternative, especially in resource-limited jurisdictions, and in scenarios where SBRT may be technically challenging.

Comparing the LUSTRE trial with similar trials, the CHISEL trial had shorter follow-up and permitted a biologically inferior CRT dose compared with the LUSTRE trial (eAppendix 7 in Supplement 2),^5,6^ This may explain the improvement in LC observed with SBRT in the CHISEL trial. The SPACE trial also observed no difference in LC for SBRT compared with CRT.

The LUSTRE trial included both central and ultracentral NSCLC, using a risk-adapted SBRT dose. Both arms were well tolerated, with limited grade 3 or higher toxic effects. Slightly higher observed toxic effects occurred with central SBRT. Late grade 3 or higher toxic effects occurred in 6 of 45 patients (13%), similar to the RTOG 0813 central SBRT trial (12.1% for 55 or 60 Gy in 5 fractions).^11^ In the LUSTRE trial, 1 patient with ultracentral NSCLC receiving SBRT developed grade 5 hemoptysis, despite meeting trial planning objectives. A phase 2 trial (HILUS trial) expressed caution using similar SBRT doses (56 Gy in 8 fractions), reporting 15% treatment-related death for ultracentral tumors.^12^ Our preliminary analysis demonstrated a 19% risk of grade 3 or higher late toxic effects using SBRT, but numbers are small, and further evaluation/dosimetric analysis is required.

### Strengths and Limitations

Trial strengths included the RTQA credentialing/review, blinded central adjudication, and multicenter contributions (eAppendix 8 in Supplement 2). Limitations beyond low accrual/statistical power were the inclusion of non–biopsy-proven NSCLC in more than half of patients (118 of 233 [51%]). While this approach reflects common practice in many institutions,^13,14^ the proportion is higher than previously reported trials. Although central radiological review of LC did not occur, clinicians were provided strict criteria to help distinguish recurrence from posttreatment fibrosis^15^ and central adjudication helped ensure confidence in the results.

## Conclusions

In summary, to our knowledge, the LUSTRE trial is the first RCT to compare lung SBRT with hypofractionated CRT that included central and ultracentral tumors. We were unable to detect a difference in LC between groups. The trial provides important prospective evidence evaluating SBRT, but further research is necessary to determine if SBRT is more effective than CRT for peripheral and central stage I NSCLC.

## Notes

Supplement 1. Trial Protocol

Supplement 2. **eAppendix 1.** Trial Sites and Investigators **eAppendix 2.** Radiation Planning Guide **eAppendix 3.** Definition of Outcomes and Censoring Rules **eAppendix 4.** Baseline Characteristics **eAppendix 5.** Cumulative Incidence of Local Failure, Patterns of Failure, and Causes of Death **eAppendix 6.** Further Toxic Effects Details **eAppendix 7.** SBRT vs CRT RCT Dose Comparison **eAppendix 8.** Recruitment Table by Center

Supplement 3. Data Sharing Statement
