# Supplementary material for: Stereotactic vs Hypofractionated Radiotherapy for Inoperable Stage I Non–Small Cell Lung Cancer: The LUSTRE Phase 3 Randomized Clinical Trial
Source: JAMA Oncol. 2024 Sep 19;10(11):1571–5. doi: 10.1001/jamaoncol.2024.3089 (PMC11413752; doi:10.1001/jamaoncol.2024.3089)
Supplement: Supplement 1. — Trial Protocol [file jamaoncol-e243089-s001.pdf]

# APPENDIX B
**Trial Protocol**

## CLINICAL TRIAL PROTOCOL

# A Randomized Trial of
# Medically Inoperable Stage I Non-Small Cell Lung Cancer Patients
# Comparing Stereotactic Body Radiotherapy
# Versus Conventional Radiotherapy
# (LUSTRE)

**Protocol Number: OCOG-2013-LUSTRE**

**Protocol Version: 6.0**

**Protocol Date: November 24, 2021**

## CO-PRINCIPAL INVESTIGATORS

**Dr. Anand Swaminath**
Juravinski Cancer Centre
Radiation Oncology
699 Concession Street,
Hamilton, ON L8V 5C2

Tel: 905-387-9495 ext. 64706
Email: swaminath@hhsc.ca

Signature

06 Dec 2021

Date

**Dr. Tim Whelan**
Juravinski Cancer Centre
Radiation Oncology
699 Concession Street,
Hamilton, ON L8V 5C2

Tel: 905-387-9711 ext. 64501
Fax: 905-575-6308
Email: **twhelan@hhsc.ca**

Signature

13 Dec 2021

Date

## CO-INVESTIGATORS

**Dr. Marcin Wierzbicki**
Juravinski Cancer Centre
Medical Physics
699 Concession Street,
[illegible]

T[illegible]
F[illegible]

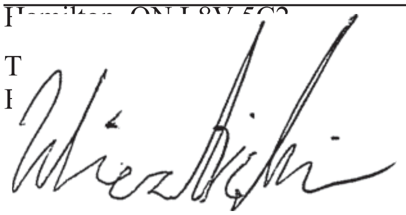

Signature

2021/12/13

Date

**Dr. Sameer Parpia**
Ontario Clinical Oncology Group
Juravinski Hospital
G Wing, 1st Floor
711 Concession Street,
Hamilton, ON L8V 1C3

Tel: 905-527-2299 ext. 42685
Fax: 905-575-2639
Email: parpia@mcmaster.ca

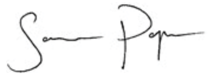

Signature

2021/12/02

Date

**SPONSOR**
**Ontario Clinical Oncology Group (OCOG)**

Dr. Jim Wright
Director, Ontario Clinical Oncology Group
McMaster University, Faculty of Health Sciences, Department of Oncology
Juravinski Hospital Research Centre, G Wing, 1st Floor
711 Concession Street
Hamilton, ON L8V 1C3

Signature

06 Dec 2021

Date

## STUDY SCHEMA

**TARGET POPULATION** satisfying both of the following:

1. Histologically confirmed T1/T2a N0 M0 NSCLC (squamous cell, adenocarcinoma, large cell carcinoma, or not specified) and CT thorax and/or PET-CT evidence ***or*** a suspicious growing nodule on serial CT.
2. Deemed medically inoperable, ***or***
   radiotherapy is preferred by the patient due to high operable risk.

**EXCLUSIONS**
Meeting any Exclusion Criteria
See Section 4.1.2

**ELIGIBLE AND CONSENTING**

**R**
**2E: 1C**

**Strata:**
1. Tumour size/stage (T1≤ 3cm vs. T2a >3-5cm)
2. Site of primary tumour (central vs. peripheral)
3. Clinical centre

**SBRT**
**(Experimental)**
48Gy/4f or 60Gy/8f
over 8-14 days
See Section 7.1

**CRT**
**(Control)**
60Gy/15f
over 3 weeks
See Section 7.2

**FOLLOW-UP**

2 weeks post end of radiotherapy,
3, 6, 9, 12, 18, 24, 30 months, 3, 4 and 5 years post randomization

**STUDY OUTCOMES**

**Primary Outcome:**
Local control (LC)

**Secondary Outcomes:**
1. Overall Survival
2. Disease-Free Survival
3. Event-Free Survival
4. Lung Cancer-Specific Survival
5. Radiation Treatment-Related Death
6. Toxicity
7. Quality of Life
8. Cost-Utility

**Planned Sample Size: 324 Patients (216 SBRT, 108 CRT)**

## LIST OF ABBREVIATIONS

| | |
|---|---|
| BED | Biological Effective Dose |
| CI | Confidence Interval |
| CMC | Coordinating and Methods Centre |
| COPD | Chronic Obstructive Pulmonary Disease |
| CRF | Case Report Form |
| CRT | Conventional Radiotherapy |
| CT | Computed Tomography |
| CTV | Clinical Target Volume |
| DFS | Disease Free Survival |
| DSMC | Data Safety Monitoring Committee |
| ECOG | Eastern Cooperative Oncology Group |
| EDC | Electronic Data Capture |
| EORTC | European Organization for Research and Treatment of Cancer |
| GCP | Good Clinical Practice |
| GTV | Gross Tumour Volume |
| Gy | Gray |
| HR | Hazard Ratio |
| ICF | Informed Consent Form |
| IMRT | Intensity Modulated Radiotherapy |
| IRIS | Interactive Registration/RandomIzation System |
| ITV | Internal Target Volume |
| LC | Local Control |
| LCSS | Lung Cancer Specific Survival |
| MRI | Magnetic Resonance Imaging |
| NCIC | National Cancer Institute of Canada |
| NCI-CTCAE | National Cancer Institute Common Terminology Criteria for Adverse Events |
| NSCLC | Non-small Cell Lung Cancer |
| OAR | Organs at Risk |
| OCOG | Ontario Clinical Oncology Group |
| ORCCID | Online Remote Collection of Clinical Information and Data |
| OS | Overall Survival |
| PET | Positron Emission Tomography |
| PTV | Planned Target Volume |
| QA | Quality Assurance |
| QALYs | Quality Adjusted Life Years |
| QI | Qualified Investigator |
| QoL | Quality of Life |
| RCT | Randomized Clinical Trial |
| REB | Research Ethics Board |
| RP | Radiation Pneumonitis |
| RT | Radiotherapy |
| RTRD | Radiation Treatment Related Death |
| SBRT | Stereotactic Body Radiotherapy |
| VMAT | Volumetric Modulated Arc Therapy |

# TABLE OF CONTENTS


STUDY SCHEMA ..... 5
*LIST OF ABBREVIATIONS* ..... 6
*TABLE OF CONTENTS* ..... 7
*1 BACKGROUND AND RATIONALE* ..... 9
*1.1. Management of NSCLC* ..... 10
*1.2. Conventional Radiation Therapy* ..... 10
*1.3. Stereotactic Body Radiotherapy* ..... 11
*1.4. Study Rationale* ..... 13
*2 STUDY OBJECTIVES* ..... 14
*2.1. General Objective* ..... 14
*2.2. Primary Objective* ..... 14
*2.3. Secondary Objectives* ..... 14
*3 STUDY DESIGN* ..... 14
*4 STUDY POPULATION* ..... 14
*4.1. Eligibility* ..... 14
*4.1.1. Inclusion Criteria* ..... 15
*4.1.2. Exclusion Criteria* ..... 15
*5 SUBJECT ENROLLMENT* ..... 15
*5.1. Randomization Procedure* ..... 15
*5.2. Stratification* ..... 16
*6 BASELINE ASSESSMENT* ..... 16
*7 STUDY TREATMENT* ..... 16
*7.1. SBRT (Experimental Arm)* ..... 16
*7.2. CRT (Control Arm)* ..... 18
*7.3. Radiation Quality Assurance* ..... 18
*8 ADJUVANT SYSTEMIC AND SUPPORTIVE THERAPY* ..... 19
*8.1. Permitted Concomitant Medication and Therapy* ..... 19
*8.2. Prohibited Concomitant Medication and Therapy* ..... 19
*8.3. Supportive Therapy* ..... 20
*9 EVALUATION DURING AND AFTER RADIOTHERAPY* ..... 20
*9.1. Follow-up Assessment* ..... 20
*9.2. Early Permanent Discontinuation of Radiotherapy* ..... 20
*9.3. Follow-up after Early Permanent Discontinuation of Radiotherapy* ..... 20
*10 CANCER RECURRENCE AND OTHER CANCER EVENTS* ..... 20
*11 STUDY OUTCOMES* ..... 21
*11.1. Primary Outcome* ..... 21
*11.2. Secondary Outcomes* ..... 21
*12 STATISTICAL CONSIDERATIONS* ..... 23
*12.1. Statistical Analysis* ..... 23
*12.2. Sample Size and Feasibility* ..... 24
*12.3. Planned Interim Analysis* ..... 25
*13 HEALTH SERVICES RESEARCH* ..... 25
*13.1. Quality of Life* ..... 25
*13.2. Economic Evaluation* ..... 25

*14 STUDY SIGNIFICANCE ..... 27*
*15 ETHICAL AND REGULATORY STANDARDS ..... 27*
*15.1. Informed Consent ..... 28*
*15.2. Research Ethics Board (REB) ..... 28*
*16 RESPONSIBILITIES OF THE INVESTIGATOR ..... 29*
*17 STUDY MONITORING AND DATA HANDLING ..... 29*
*17.1. Data Collection Method ..... 29*
*17.2. Source Document Requirements ..... 29*
*17.3. Retention of Study Records ..... 30*
*18 CONFIDENTIALITY ..... 30*
*19 CLINICAL TRIAL PROTOCOL AMENDMENTS ..... 30*
*20 STUDY ORGANIZATION ..... 30*
*20.1. Steering Committee ..... 30*
*20.2. Data Safety Monitoring Committee ..... 31*
*20.3. Study Coordination ..... 31*
*20.4. Central Adjudication Committee ..... 31*
*21 SCIENTIFIC REPORTING AND PUBLICATION ..... 32*
*22 REFERENCES ..... 33*
*APPENDIX I: SCHEDULE OF STUDY ASSESSMENTS AND EVALUATIONS ..... 37*
*APPENDIX II: TNM CANCER STAGING ..... 38*
*APPENDIX III: ECOG PERFORMANCE STATUS ..... 40*
*APPENDIX IV: CHARLSON COMORBIDITY INDEX ..... 41*
*APPENDIX V. QUALITY OF LIFE QUESTIONNAIRES ..... 42*
*APPENDIX VI: EQ-5D QUESTIONNAIRE ..... 45*

# 1 BACKGROUND and RATIONALE

Surgery is considered the standard of care for stage I (T1/T2a N0), non-small cell lung cancer (NSCLC). The expected survival rate following surgical resection is approximately 60 to 80% at 5 years.[1,2] However, approximately 20-25% of patients are deemed medically inoperable due to significant comorbidities, including severe chronic obstructive pulmonary disease (COPD) and cardiovascular disease.[3,4] In this group of patients, radiotherapy (RT) is the preferred treatment modality. In the past, RT was planned using conventional techniques (2D x-ray imaging), large treatment margins, and simple beam geometries, and was delivered using standard linear accelerators without correction for respiratory motion and daily image verification.[5] The treatment was fractionated with prolonged courses of 60-66 Gray (Gy) in fractions of 2Gy each over 6-6.5 weeks.[6] Case series using this approach reported 3-year rates of local control (LC) from 40 to 50% and overall survival (OS) from 17 to 55%.[5-8] Cancer progression was most frequently seen locally (40%) with distant metastases occurring in approximately 25% of patients. The poor outcomes were attributed primarily to an inability to cure the primary tumour as well as competing risks from other comorbidities.

Currently in Canada, ***conventional radiotherapy*** (CRT) for stage I NSCLC is most often delivered using a larger dose per fraction (hypofractionation) in a shorter (accelerated) time period. This is based on improving the convenience of treatment for patients and on the radiobiological principles that larger doses per fraction are more effective in causing cancer cell death and shorter treatments prevent repopulation of the tumour. Studies from the Netherlands, the US and Canada using doses of 48-70Gy in 3-4Gy fractions support this approach for lung cancer with improved LC (freedom from local recurrence) rates of 70 to 80% and OS rates of 35 to 50% at 3 years.[9-11] Minimal acute and long-term toxicity have been observed. As a result, conventional therapy in Canada is now most often delivered using 50-60Gy in 12-20 fractions of 3-4Gy fractions over 3-4 weeks.

Recently with the advent of major innovations in RT technology, investigators have applied ***stereotactic body radiotherapy*** (SBRT) to the treatment of inoperable patients with early stage NSCLC.[12] SBRT is a noninvasive ablative treatment derived from principles of cranial stereotactic radiosurgery. It delivers large doses of radiation per fraction (typically 7.5-18Gy) in a limited number of treatments (3-8 fractions), to sites outside the brain.[13] As a result of significant innovations and improvements in RT planning and delivery, SBRT has been applied to tumours such as lung cancer that move with internal body movement. Case series and prospective phase I-II studies have reported high rates of LC (87 to 95%) and OS (65 to 76%) at 2 years.[14-20] These studies however are small with limited follow up, and one study reported significant long term toxicity (fatal hemoptysis and pneumonia) when very high doses per fraction (20Gy) were used to treat centrally located tumours.[21] Based on these observations, SBRT when carefully delivered is an attractive treatment for patients with medically inoperable stage I NSCLC. It is more convenient for patients and it is predicted that such treatment will result in improved LC and potentially OS. However, in contradistinction to cranial radiosurgery, as yet, no randomized trials have shown thatSBRT improves patient outcomes compared to CRT.[22,23]

## 1.1. Management of NSCLC

Patients who present with NSCLC undergo staging to determine the extent of the disease; computed tomography (CT) thorax for intrathoracic spread; CT abdomen for spread to liver/adrenals; and positron emission tomography (PET) for bone and other distant sites.[24] In some instances, patients undergo mediastinoscopy to determine whether the tumour has metastasized to the mediastinum. Patients with stage I disease (i.e. tumours $\leq$ 5cm), with no cancer in the lung hilum and mediastinum undergo surgery. Approximately 25% of patients with early NSCLC cannot undergo surgery because of comorbid disease e.g. COPD.[3,4] These patients are usually treated with RT.

## 1.2. Conventional Radiation Therapy

There are no randomized trials of CRT for stage I NSCLC. A large systematic review of CRT in medically inoperable stage I NSCLC was performed by Qiao et al[5]. Investigators reviewed the English literature from 1988-2000 for studies of CRT in medically inoperable patients. They identified 18 studies, the majority of which were retrospective case series; only 5 studies included more than 100 patients. Different radiation therapy regimens were used ranging from 48 to 69.6Gy in 1.2-4Gy fractions. Investigators reported that the most common reason for treatment failure were high rates of local recurrence ranging from 10-70% (median 40%). The biological effects of differing total radiation doses and fraction sizes can be predicted by radiobiological models. One common model is the Biological Effective Dose (BED)[25], which estimates the effective dose for both tumour control (BED10) and toxicity (BED2). In this systematic review, higher doses of radiation as estimated by BED were associated with improved rates of LC. Of note, improved LC rates were associated with an increased cause-specific and OS. Although poorly reported, the rate of radiation pneumonitis (RP) /pulmonary fibrosis was low (<5%), and no radiation treatment-related deaths (RTRD) were observed. Systematic reviews conducted previously by Sibley[8] and Rowell[7] reported similar results.

Given that the review by Qiao et al was performed more than 10 years ago, we reviewed hypofractionated CRT regimens currently in use for stage I NSCLC (**Table 1**). Seven studies were identified; 4 from Canada and 1 each from the UK, the Netherlands, and the US. An early Canadian study by Faria et al used hypofractionated doses of 52Gy in 15 fractions in 31 patients and reported 2 year LC of 76% with no severe toxicity.[26] In a more recent study from Canada, Soliman and Cheung et al evaluated doses of 48-60Gy in 12-15 fractions in 118 patients and reported 2 and 5-year rates for LC of 76% and 70% and OS of 51% and 23%, respectively.[11] Acute toxicity was limited, 5 patients required treatment for RP and only 1 death was attributed to treatment. This led to the recently completed NCIC BR.25, a Canadian multi-centre, prospective cohort study of hypofractionated CRT using 60Gy in 15 fractions in 80 patients.[27] At a median follow-up of 3 years, the 2-year LC rate was 88%. Reports of acute and long-term toxicity were limited; only six grade 4 (resulting in hospitalization) or 5 (TRM) toxicities were observed. Only one death (massive hemoptysis) was attributed to therapy. Based on these studies reporting improved LC in comparison to historical controls, and the attractiveness of a short schedule with limited toxicity, 60Gy in 15 fractions is now the most commonly used radiation regimen in Canada for the treatment of medically inoperable stage I NSCLC.

**Table 1**
**Prospective Studies in Conventional Accelerated Hypofractionated Radiotherapy in Stage I NSCLC***

| | | | | | Local Control (%) | | Overall Survival (%) | | Toxicity (%) | |
|---|---|---|---|---|---|---|---|---|---|---|
| Study | Country | N | Median F/up (months) | Dose | At 2 years | At 5 years | At 2 years | At 3 years | Grade **≥ 3+** | Grade **5++** |
| Slotman *et al* (1996) [10] | Netherlands | 31 | -- | 48Gy/ 12 frac | -- | -- | -- | 42 | -- | -- |
| Cheung *et al* (2000) | Canada | 102 | 86 | 52.5Gy/ 20 frac | -- | -- | -- | 35 | 3 to 5 | 0 |
| Lester *et al* (2004) | UK | 135 | 48 | 50-55Gy/ 15-20 frac | -- | -- | 48 | 32 | 0 | 0 |
| Faria *et al* (2005) [26] | Canada | 31 | 29 | 52Gy/ 15 frac | 76 | -- | 56 | -- | 0 | 0 |
| Bogart *et al* (2009) [9] | US | 39 | 53 | 70Gy/ 17-29 frac | -- | -- | 50 | -- | 0.25 | 0 |
| Soliman *et al* (2011) [11] | Canada | 118 | 24 | 48-60Gy/ 12-15 frac | 76 | 70 | 51 | -- | 5 | 0.8 |
| Cheung *et al* (2012) [27] | Canada | 80 | -- | 60Gy/ 15 frac | 88 | -- | -- | -- | 8 | 1.3 |

* Indicates not reported; + Grade 3 toxicity (requiring medication); ++ Grade 5 toxicity (resulting in mortality)

## 1.3. Stereotactic Body Radiotherapy

SBRT is a non-invasive ablative treatment derived from principles of cranial stereotactic radiosurgery. Radiosurgery delivers very high doses of radiation per fraction in a limited number of treatments with the goal of destroying the cancer and avoiding the invasiveness of surgery. Extracranial radiosurgery, or SBRT, involves sophisticated techniques to account for tumour motion and respiration including 4-dimensional CT planning, respiratory gating and real-time tracking of the tumour as it moves.[28] Highly conformal techniques such as intensity modulated radiotherapy (IMRT) and volumetric modulated arc therapy (VMAT) can improve the ability to deliver high doses while sparing normal tissues.[29] Reproducible patient immobilization, as well as daily image guidance to localize and correct for any changes in tumour position have increased treatment accuracy.[30]

Two systematic reviews have been published on studies of SBRT. Zhang et al reviewed all studies of SBRT involving 20 patients or more published between 2001 and June 2010.[31] They identified 34 studies with a total of 2,500 patients, with the majority of studies published after 2008. All studies were observational, average size was 76 patients and only 5 studies had more than 100 patients. Average median follow-up was only 27 months with 1 study > 48 months. Pooled data for 2-year LC and OS were 92.3% (90.7-94%), and 63.2% (60.1-65.6%) respectively. The 5-year results (from a limited number of studies) were 76.4% (71.2-80.8%) and 36.0% (22.9-51.5%), respectively. BED10 values > 106Gy were associated with improved LC and survival compared to lower doses. Overall, only 142 of

2587 (5.5%) patients were reported to have moderate-severe toxicity. The incidence was less in patients who received a BED10 < 146Gy. There are limitations to any inferences between dose and outcome because of the uncontrolled study designs.

A second systematic review pooled results from 11 observational studies of SBRT and compared them to 11 studies of CRT.[32] Details on LC were not reported but SBRT was associated with higher rates of 2-year OS in comparison to CRT; median 70% (63-73%) vs. 53% (46-60%); p<0.001. TRM was uncommon in both groups; 0.1% for CRT and 0.7% for SBRT. All SBRT related deaths (n=6) were from one trial, which used a high BED10 (180Gy) to treat central tumours. No TRM was reported for SBRT studies that used a lower BED10. There are limitations to the comparison between SBRT and CRT because these studies were not randomized, assessments of outcomes and toxicity were variable, and follow-up was limited.

In addition to these reviews, we also report results from prospective phase I and II studies with >30 patients. We identified six studies, the majority of which were small in size and with limited follow-up. The largest study was from the Netherlands where 206 patients were treated with 60Gy in 3-8 fractions.[33] Median follow-up was 12 months, with 2-year LC of 93% and OS of 65%. Six patients (3%) were reported to have grade 3 (symptomatic requiring steroids) or higher RP, and there was no TRM. The largest Canadian experience was from Princess Margaret Hospital in Toronto, where 108 patients were treated with 48Gy in 4 fractions or 54Gy in 3 fractions for peripherally located tumours, and 60Gy in 8 fractions for central lesions.[20] Median follow-up was 19 months. LC was 92% at 1-year and 89% at 4-years with no grade 4 or 5 toxicities. A multi-centre prospective study was reported by the RTOG using 54Gy in 3 fractions for peripheral lesions in 55 medically inoperable patients. After a median follow-up of 34 months, investigators reported a 3-year LC of 98% and OS of 56%. Grade 3 or higher pulmonary toxicity was observed in 9 patients (16%) and there was no RTRD.[18]

SBRT has been associated with unique toxicities not previously observed with CRT. One of the earliest prospective trials from Indiana of 60Gy in 3 fractions (BED10=180, BED2=660) reported 6 deaths in 70 patients attributable to therapy; causes of death were pneumonia (4), pericardial effusion (1) and hemoptysis (1).[21] A multivariate analysis showed that tumours located in the central or hilar regions were significantly predictive of severe toxicity (p=0.04). The high risk of death was attributed to the high BED used in this study. Studies that have used lower doses of SBRT, such as 48Gy in 4 fractions of 12Gy (BED10=106, BED2=336)[17] or 60Gy in 8 fractions (BED10=105, BED2=285)[16,34] have not been associated with such severe toxicities as demonstrated in systematic reviews previously described. Haasbeek et al reported on 63 patients treated with a dose of 60Gy in 8 fractions for central lesions.[34] There were only 3 grade 3 toxicities and no RTRD. LC was similar to more aggressive doses, with a rate of 93% at 2 and 5 years.

The conclusion from our review is that SBRT appears very promising, but it can be associated with severe toxicity when appropriate techniques are not employed. All available studies are still observational in nature with no formal controls. The majority of studies are small, patients may have been highly selected and follow-up is limited. In addition, toxicity

was not measured in a consistent way across all studies. Based on this review, we feel that further comparative evaluation is necessary.

## 1.4. Study Rationale

Increases in reported rates of LC along with a shorter radiation schedule using SBRT have led to widespread interest in the use of this treatment for medically inoperable NSCLC. It is currently being widely promoted throughout North America and Europe by radiation oncologists based on limited data from a number of non-randomized pilot studies. There is an increasing concern that this new technology like other new costly radiation technologies e.g. accelerated partial breast irradiation for early stage breast cancer or interstitial brachytherapy for prostate cancer will be adopted into practice based on financial and other incentives without proper evaluation. While SBRT is attractive, it does have its limitations. SBRT is technologically intensive requiring specialized CT scanning and linear accelerator equipment. It also requires specialized training for radiation physicists, therapists and oncologists. Although SBRT is delivered in fewer fractions than CRT, it requires special quality assurance (QA) procedures and each treatment can last up to 60 minutes compared to CRT, which requires only 10-15 minutes for each treatment. Consequently, the overall machine time and associated costs required are potentially substantially higher for SBRT.[35]

In a recent survey of American radiation oncologists, 65% indicated that they had adopted SBRT and of these 89% were using it to treat NSCLC. Reported reasons for adopting SBRT were to take advantage of the biological higher dose of radiation and to gain a competitive advantage in the marketplace.[36] In contrast to other countries; Canada has been slower to adopt SBRT. A recent survey reported that only 13 out of 32 RT centres in Canada were using SBRT for the treatment of selected patients with NSCLC.[37] The reasons for not adopting SBRT by the 18 non-user centres were increased resources, clinical and physics expertise required and lack of level I evidence on the efficacy of treatment. CRT continues to be more commonly used in Canada as data support adequate local control, increased resources are not required, and such treatment remains within the scope of most radiation oncologists.

There are currently two small randomized phase II trials evaluating SBRT in comparison to CRT. TROG-0902 is an Australian/New Zealand trial comparing a standard arm of 60-66Gy in 2Gy fractions over 6-6.5 weeks (with concurrent chemotherapy, if desired) vs. SBRT of 54Gy in 3 fractions of 18Gy each.[38] The sample size is 100 patients. A Scandinavian study, the SPACE trial, is comparing CRT of 70Gy in 35 fractions over 7 weeks vs. SBRT of 45Gy in 3 fractions of 15Gy each. The sample size is 100 patients. Both trials have limitations: (1) they are not comparing SBRT to hypofractionation schedules used in Canada; (2) the SBRT fractionation regimens are not commonly used in Canada; and (3) the trials are underpowered to address the impact of SBRT on LC and toxicity and other important secondary outcomes like OS, quality of life and cost-utility.

Currently there is increasing interest in the use of SBRT for NSCLC in Canada. There is a real danger that if a randomized trial is not initiated soon, the opportunity will be missed to adequately evaluate this promising new technology. Our goal is to perform a randomized trial comparing SBRT to CRT delivered in 3 weeks consistent with Canadian practice. The trial will be adequately powered to look at the impact of SBRT on LC and RTRD. In

addition, we will also evaluate the impact of treatment on quality of life and cost-effectiveness. If such a study showed that SBRT improved LC with limited toxicity and at an acceptable cost, it would lead to widespread adoption as the standard of care in Canada. On the other hand, if such a study proved not to result in improved patient outcomes, it would support a policy against widespread adoption in Canada of a more costly, potentially less effective and more toxic treatment.

# 2 STUDY OBJECTIVES

## 2.1. General Objective

To determine if SBRT is more effective than CRT for medically inoperable patients with stage I NSCLC.

## 2.2. Primary Objective

To determine if SBRT as compared to CRT improves local control (LC) for medically inoperable patients with stage I NSCLC.

## 2.3. Secondary Objectives

In these two treatment approaches:

- To compare overall survival.
- To compare disease-free survival.
- To compare event-free survival.
- To compare lung cancer-specific survival.
- To compare radiation treatment-related deaths.
- To evaluate the toxicity effects.
- To evaluate the associated quality of life.
- To evaluate the cost-utility.

# 3 STUDY DESIGN

A multi-centre randomized controlled open-label trial in medically inoperable patients with biopsy-proven early stage NSCLC. Eligible and consenting patients will be randomly allocated to receive SBRT or CRT in a 2:1 ratio. Radiotherapy will be administered as soon as possible following randomization and subjects will be followed for 5 years post-randomization for cancer recurrence, toxicity and survival. The primary outcome is LC. The trial will be conducted at 16-20 clinical centres throughout Canada.

# 4 STUDY POPULATION

## 4.1. Eligibility

Eligibility status must be confirmed by the local investigator or designate prior to enrollment. A PET-CT must be performed within 3 months of randomization (then a CT thorax within 8 weeks is not necessary). If PET-CT is not performed within 3 months then an up-to-date CT thorax within 8 weeks is acceptable. There must be no change in clinical stage. Brain imaging investigations, performed within 3 months prior to randomization, are preferred but not required. Pulmonary Function Tests can be done anytime prior to randomization. It is

important that no exceptions be made to the eligibility criteria. Questions related to eligibility requirements or specific criteria must be discussed with OCOG prior to enrollment.

### 4.1.1. Inclusion Criteria

For inclusion in this study, patients must fulfill ***both*** of the following criteria:

1. T1/T2a N0 M0 NSCLC, either by: (a) histological confirmation (squamous cell, adenocarcinoma, large cell carcinoma, or not specified) and CT thorax and/or PET-CT evidence, or (b) a suspicious growing nodule on serial CT imaging, with malignant PET FDG avidity, for which a biopsy would be extremely risky (*see **Appendix II** on TNM Staging*).
2. Deemed medically inoperable (as reviewed by a thoracic surgeon and defined as surgically resectable but, because of underlying physiological medical problems [e.g. COPD, heart disease], surgery is contraindicated)
   ***or***
   Radiotherapy is preferred by the patient due to high operable risk.

### 4.1.2. Exclusion Criteria

Patients who satisfy any of the following exclusion criteria are NOT eligible for this study:

1. Less than 18 years of age.
2. ECOG performance status 3 or higher (*see **Appendix III***).
3. Prior invasive malignancy within the past 3 years (excluding non-melanomatous skin cancer).
4. History of ataxia telangiectasia.
5. Previous RT in the vicinity of the tumour, such that significant overlap could occur.
6. Previous pneumonectomy with Stage I lung cancer in the remaining lung.
7. Diagnosis of idiopathic pulmonary fibrosis and/or interstitial lung disease
8. Planned for other anticancer therapy (chemotherapy, biological targeted therapy).
9. Female, who is currently pregnant or lactating.
10. Geographic inaccessibility for follow-up.
11. Unable to provide informed consent.

## 5 SUBJECT ENROLLMENT

### 5.1. Randomization Procedure

Patients will be allocated in a 2:1 fashion (SBRT:CRT) to one of two treatment groups:

| Treatment Group | Intervention | |
|---|---|---|
| 1 | SBRT | Experimental arm |
| 2 | CRT | Standard arm |

Randomization will be conducted centrally by the OCOG Coordinating and Methods Centre (CMC) located at the Juravinski Hospital in Hamilton, Ontario. After confirmation of patient eligibility and documentation of written informed consent, clinical centres will complete randomization by accessing the CMC's web-based Interactive Registration and Randomization System (IRIS). Randomization will be performed according to a prescribed computer generated schedule.

### 5.2. Stratification

Stratification will be utilized prior to randomization to ensure balance between treatment arms for factors that might influence the primary outcome. Therefore, eligible consenting patients will be stratified by:

(1) Tumour size[ǂ]/stage (T1 ≤ 3cm vs. T2a > 3-5cm)

(2) Site of primary tumour (central* vs. peripheral)

(3) Clinical centre.

ǂ For tumour size measurement at stratification, use CT if within 3 months, otherwise use PET-CT

* Central tumours are defined as either within 1 cm of mediastinum or 2 cm within the zone of the proximal bronchial tree on staging CT or PET-CT.

## 6 BASELINE ASSESSMENT

Refer to ***Appendix I*** (*Schedule of Study Assessments and Evaluations).*

The Baseline assessment includes the collection of patient demographics, height and weight, documentation of cancer TNM staging (CT thorax and/or PET-CT, and CT/MRI brain, if performed), ECOG performance status, pulmonary function tests (PFTs), baseline symptom assessment and comorbidity status using the Charlson comorbidity index (***Appendix IV***).

The quality of life questionnaires (EORTC QLQ-C30 and LC-13; ***Appendix V***) will be completed by all patients prior to randomization, and then for a period of 2 years.

In addition, study patients will be asked to complete the EuroQol EQ-5D Health questionnaire (***Appendix VI***) prior to randomization and then for a period of 3 years to assess health utilities.

## 7 STUDY TREATMENT

Radiation therapy will be administered as soon as possible following enrolment (within 3 weeks post randomization). The following sections are an overview. Details regarding radiation planning will be included in a separate radiation planning guide.

### 7.1. SBRT (Experimental Arm)

(1) **Aim and Technique:** The aim is to cover the gross tumour volume (GTV) with an adequate margin for respiratory motion (internal target volume, ITV), and patient set-up (planning target volume, PTV). CT simulation is required, IV contrast is not mandatory. A 4-dimensional (4D) CT scan is required in order to account for

respiratory motion. No mediastinal/hilar nodal radiation is to be performed. CT planning will be executed, with multiple-beam conformal or IMRT, VMAT, or robotic radiosurgery techniques, in order to achieve adequate conformality around the target, and avoidance of normal tissues. Special consideration will be made to account for the effect of internal organ motion (e.g. breathing) on target positioning and reproducibility.

(2) **Position:** Patients will be treated in the supine position and while free-breathing, or with controlled breathing techniques such as abdominal compression, respiratory gating, or active breath-holding techniques.

(3) **Treatment Volumes:** The GTV as defined as the visible tumour seen on the planning CT simulation is required. No clinical target volume (CTV) delineation for microscopic disease will be performed. The ITV accounts for changes in tumour position due to respiration and is not required when breath-holding techniques are used (i.e. the ITV is the same as the GTV). The PTV will be an isotropic expansion of 0.5 cm around the ITV.

(4) **Energy:** A linear accelerator capable of 4-10 MV energy, equipped with a multi-leaf collimator of at most 10 mm leaf width should be used. *Cyberknife*, a robotic linear accelerator of 6 MV is permitted.

*(5)* **Field Arrangement:** 3-dimensional coplanar and non-coplanar beam geometries should be used to cover the PTV adequately. Other alternatives include using IMRT, VMAT, or robotic radiosurgery techniques.

(6) **Dose-Fractionation:** The prescription dose is 48Gy in 4 fractions of 12Gy for peripheral tumours, delivered every other day. For lesions located within 1 cm of the heart/great vessels or within 2 cm of the proximal bronchial tree, a risk-adapted dose of 60Gy in 8 daily fractions of 7.5Gy will be prescribed. The proximal bronchial tree is defined as the distal 2 cm of trachea, both right and left mainstem bronchi, the right and left upper lobe bronchi, bronchus intermedius, left lingular bronchus, right middle and lower lobe bronchus, and left lower lobe bronchus. Treatment will be delivered with a minimum of 8 days to a maximum of 14 days from the start to finish. PTV coverage will be confirmed using dose-volume histograms.

(7) **Normal Tissue Restrictions:** Critical organs at risk (OARs) include the trachea, proximal bronchial tree, esophagus, lungs, spinal cord, brachial plexus, heart, great vessels, stomach and ribs. In cases of overlap between PTV and OARs, dose constraints on OARs will be considered higher priority (with the exception of rib dose).

(8) **Treatment Verification:** Patients treated on linear accelerators will require daily cone-beam CT imaging with dedicated matching to bone and soft tissue (ITV) prior to each fraction. Patients treated with robotic radiosurgery will require tumour tracking with fiducial marker placement prior to treatment.

### 7.2. CRT (Control Arm)

(1) **Aim and Technique:** The aim is to cover the GTV with an adequate margin for respiratory motion (ITV), and patient set-up (PTV). CT simulation is required. A 4-dimensional CT is permissible but not required. No mediastinal/hilar nodal radiation is to be performed. CT planning is to be executed, with 3D conformal RT techniques (IMRT is permitted if centres are capable of planning using this technique).

(2) **Position:** Patients will be treated in the supine position, with both arms raised above their head. Immobilization using a chest board is encouraged. Strategies to limit internal organ motion are not required.

(3) **Treatment Volumes:** The GTV is defined as the visible tumour as seen on the planning CT simulation. There will be no additional CTV. The ITV is defined using the 4D-CT if available. The PTV will be a minimum isotropic 0.5 cm expansion around the ITV. If respiratory motion is not accounted for (no 4DCT/ITV), PTV margins should be larger (minimum of 1 cm).

(4) **Energy:** A linear accelerator capable of 4-10 MV energy should be used.

(5) **Field Arrangement:** At least 3 beams using conformal techniques should be used to cover the PTV adequately. IMRT techniques can be used.

(6) **Dose-Fractionation:** The prescription dose is 60Gy in 15 fractions, 4Gy per fraction delivered daily (Monday-Friday) for 3 weeks. PTV coverage will be confirmed using DVHs.

(7) **Normal Tissue Restrictions:** OARs are similar as in SBRT, but dose constraints are different. In cases of overlap between PTV and OARs, dose constraints on OARs will be considered higher priority (with the exception of rib dose).

(8) **Treatment Verification:** Daily portal films or cone beam CT images will be taken for purposes of image verification.

### 7.3. Radiation Quality Assurance

To maintain a high degree of compliance with the protocol, to avoid contamination between treatment arms and to assure adequate safety in the delivery of SBRT, several well-established Quality Assurance (QA) processes will be incorporated in this trial.

Prior to the start of the trial, a central Radiotherapy Review Committee will be established at the Juravinski Cancer Centre in Hamilton, Ontario. The Radiotherapy Central Review Committee will consist of two radiation oncologists, a radiation therapist, and a medical physicist. All radiotherapy plans will be reviewed to establish consistency. This Committee will be responsible for developing a radiation planning guide.

**The QA process includes:**

(1) **Clinical Centre Survey:** Participating clinical centres will be surveyed regarding their practice for measuring absorbed dose, machine calibration, and treatment facilities to ensure adequate technical resources to deliver SBRT, as well as for

highly precise treatment planning and verification. It is a requirement that each centre successfully completes a plan verification experiment using the Imaging and Radiation Oncology Core (IROC), formerly Radiological Physics Center (RPC) Thorax-Lung phantom. Centres may have already completed this for other lung SBRT protocols. Confirmation of this will be required.

(2) **Radiation Planning Guide:** A planning guide will provide details for each technique, how patients should be positioned, planned and treated. It will provide contouring, planning and treatment delivery guidelines.

(3) **Centre Accreditation:** One radiation oncologist at each centre will be designated as the "primary radiation oncologist" (indicated in the Clinical Centre Survey). Each clinical centre will be provided with the Radiation Planning Guide and CT images for two training cases. The primary radiation oncologist will contour the targets (GTV, ITV, PTV) and OARs. Each centre will then plan the two cases (one central and one peripheral tumour) with the experimental and control techniques (four plans in total). These plans will be reviewed centrally to confirm protocol requirements are met. A Radiotherapy Review Manual will provide submission instructions.

(4) **Real-Time and Final Review:** Following accreditation, clinical centres will be required to submit electronic treatment plans for approximately 6 cases (first 4 SBRT and first 2 CRT cases) prior to the start of treatment for real-time review by the Central Review Committee.

Electronic submission will be in DICOM-RT format with details of contours, DVH, and dose distribution and prescription. A PDF (or equivalent) of the anonymized treatment plan including details regarding beam energies, angles and shielding are to be submitted as well. Plans are to be sent within 3 working days prior to the start of therapy to allow time for central review to ensure compliance with the protocol and allow time to amend the plan if any deviations are found. Any major deviations will be recorded by the Central Review Committee and the treatment centre will be requested to make changes to the plans before treatment is started. Centres will continue to submit plans for real-time review until 4 SBRT cases are submitted consecutively without major deviations. Following this process, all treatment plans for both SBRT and CRT will be sent for final review, which will occur within 4 weeks of treatment start.[39] A Radiotherapy Review Manual will provide submission instructions.

# 8 ADJUVANT SYSTEMIC and SUPPORTIVE THERAPY

## 8.1. Permitted Concomitant Medication and Therapy

Use of dexamethasone during radiotherapy is permitted, at the discretion of the treating oncologist.

## 8.2. Prohibited Concomitant Medication and Therapy

No chemotherapy or targeted therapies are to be given before, during or after protocol treatment with the exception of anti-neoplastic agents such as methotrexate used for

inflammatory conditions such as lupus and rheumatoid arthritis. These may be taken before and after but still not during treatment. In cases of recurrent disease the use of systemic therapy is at the discretion of the treating oncologist.

### 8.3. Supportive Therapy

There is no planned supportive therapy during protocol treatment. However, therapy used to aid in subject comfort and symptoms during treatment (pain medications, inhalers) are permitted.

## 9 EVALUATION DURING and AFTER RADIOTHERAPY

Refer to *Appendix I*: *Schedule of Study Assessments and Evaluations*

### 9.1. Follow-up Assessment

Subjects will be assessed for toxicity and survival at 2 weeks <u>post radiotherapy</u>, and they will be assessed for recurrence (CT thorax), toxicity and survival at 3, 6, 9, 12, 18, 24 and 30 months and at 3, 4 and 5 years <u>post randomization</u>, and at any unscheduled visits.

The quality of life questionnaires (EORTC QLQ-C30 and LC-13) and the health utilities questionnaire (EuroQol EQ-5D), in addition to having been completed at baseline, should be completed by the subject at 2 weeks post radiotherapy, and at 3, 6, 9, 12, 18 and 24 months post randomization. The EQ-5D will also be completed at 30 months and 3 years post randomization.

### 9.2. Early Permanent Discontinuation of Radiotherapy

All definitive treatment discontinuation should be recorded by the Investigator, along with reasons for discontinuation.

#### 9.2.1. Criteria for Early Permanent Discontinuation of Radiotherapy

Study treatment will be stopped prematurely and permanently if any of the following events occur or are diagnosed:

a) Severe acute toxicity requiring hospitalization.
b) Significant change in clinical status due to comorbid illness such that the subject is unable to continue with protocol treatment.

### 9.3. Follow-up after Early Permanent Discontinuation of Radiotherapy

Subjects who meet the criteria for early permanent discontinuation of radiotherapy and do not receive further protocol treatment will continue to be followed for cancer recurrence. Efforts should be made to continue the follow-up schedule for survival unless the subject withdraws their consent from collection of data beyond the point of withdrawal from the protocol treatment.

## 10 CANCER RECURRENCE and OTHER CANCER EVENTS

At the time of first cancer recurrence, all subjects will be fully staged with a clinical exam, CT abdomen/thorax, CT/MRI brain and/or FDG PET-CT. Site of recurrence will be recorded as will treatment at time of recurrence. Treatment of recurrence is at the discretion

of the treating oncologist and may include further radiotherapy, chemotherapy, targeted therapy, surgery, or supportive care. Subjects will continue to be followed for the primary outcome of LC.

# 11 STUDY OUTCOMES

## 11.1. Primary Outcome

**Local Control (LC)**, the primary efficacy outcome for each subject, is defined as the absence of local recurrence during the study period. Operationally, this is the time from randomization to (a) primary tumour failure or (b) marginal failure[18]. Subjects without local recurrence will be censored at the earliest date of last follow-up or death.

LC will be assessed according to current recommended guidelines for the evaluation of LC following lung RT similar to that used for BR.25[27], and from a recent review of lung parenchymal changes following SBRT.

### 11.1.1 Primary Tumour Failure

Baseline tumour measurements will be performed at the time of the planning CT. The longest diameter (LD) will be recorded. Following therapy, an enlargement of the LD as seen on $\geq 2$ serial CT examinations (at least 3 months apart) of at least 20% from the smallest size, with a concomitant absolute increase of at least 5 mm will be scored as suspicious for primary tumour failure.

### 11.1.2 Marginal Failure

Marginal failure refers to the appearance of a new growing lesion within 1 cm of the PTV after protocol treatment. Criteria to define marginal failure include an enlargement of the LD as seen on $\geq 2$ serial CT examinations of at least 20% from the initial size, with a concomitant absolute increase of at least 5 mm.

The date of local recurrence is the date of the first serial CT.

### 11.1.3 Other Suspicious Findings

Other suspicious findings to distinguish local recurrence from normal radiological changes can include loss of air bronchograms, loss of linear margin, increased bulging margin, and mass-like fibrosis. In these cases, histological evidence of local recurrence is preferred. In situations where a biopsy is not possible or deemed to be high risk, an FDG-PET may be obtained to assess response.[42] A standardized uptake value (SUV) $\geq$ 5, or an increase in metabolic uptake of 25% compared to initial tumour SUV, will be scored as suspicious for a recurrence.

## 11.2. Secondary Outcomes

Secondary outcomes include the following:

(1) **Overall Survival (OS)**

OS is defined as the time from randomization to death from any cause. Alive subjects will be censored on the date of last follow-up.

**(2) Disease-Free Survival (DFS)**

DFS is defined as the time from randomization to the earliest recurrence of disease adjudicated as: local, ipsilateral same lobe of the lung, regional (in hilum, mediastinum, or supraclavicular nodal regions), or distant (metastases involving the liver, CNS, ipsilateral other lobe of the lung, contralateral lung, bone, etc.), or to death attributed to lung cancer. Disease-free subjects will be censored on the date of last follow-up, or at the time of non-lung cancer-related death.

**(3) Event-Free Survival (EFS)**

EFS is defined as the time from randomization to the earliest documented recurrent disease or death from any cause. Event-free subjects will be censored on the date of last follow-up.

**(4) Lung Cancer-Specific Survival (LCSS)**

LCSS is defined as the time from randomization to death attributable to lung cancer. Subjects found to have evidence of local progression or regional/distant recurrence prior to death will be deemed a lung cancer-specific death. Alive subjects will be censored on the date of last follow-up, or at the time of non-lung cancer-related death.

**NOTE: All recurrences and deaths will be adjudicated independently with supportive documentation (e.g. X-rays, pathology reports, relevant clinic notes, death notes) by a Central Adjudication Committee (Section 20.4).**

**(5) Radiation Treatment-Related Death (RTRD)**

Despite interest in SBRT, there remains a concern that it can be delivered safely. It will be important to demonstrate that RTRD, although uncommon, is not increased in order to achieve widespread acceptance of SBRT.

RTRD will be defined as death, occurring between 1 to 12 months following treatment, and caused directly by radiation toxicity attributed to either: (a) catastrophic hemorrhage with severe blood loss and hypotension, or to (b) severe radiation pneumonitis, detected using classic X-ray/CT; and leading to death.

**NOTE: All deaths attributed to radiation toxicity will be adjudicated independently with supportive documentation (e.g. X-rays, pathology reports, relevant clinic notes, death notes) by a Central Adjudication Committee (Section 20.4).**

**(6) Toxicity**

Acute and late toxicity, deemed attributable to radiation, will be assessed and graded using the NCI Common Terminology Criteria for Adverse Events (NCI CTCAE) version 4.03.

**Acute Toxicity** refers to any side effect occurring during the 3 months following randomization. For the purposes of this study, acute toxicity will be formally assessed at the 2 Week Post-Radiotherapy and 3 months post randomization visit; however, any unscheduled visits for toxicity assessment up until 3 months post

randomization will be scored as acute. Common examples of acute toxicity include fatigue, dyspnea, dermatitis and esophagitis.

**Late Toxicity** refers to any side effect occurring beyond the 3-month visit. This will be assessed at every follow-up visit beginning at 6 months, and up to 5 years, post randomization. Common examples of late toxicity include radiation pneumonitis, chest wall pain, esophagitis, dyspnea and rib fracture. Rare side effects can include broncho-pulmonary hemorrhage and atelectasis/collapse secondary to airway necrosis.

**(7) Quality of Life (QoL)**

An important aim of this study is to determine whether there is any difference in the QoL that may occur between the two different radiation treatment approaches during the 2 years post-randomization. Impact on QoL will be assessed with the European Organization for Research in Treatment of Cancer (EORTC) QoL Questionnaire Core 30 (QLQ-C30)[40] and the Lung Cancer-specific Module 13 (LC-13)[41]. The QLQ-C30/LC-13 instrument measures the impact of lung cancer and its treatment on the physical, emotional, functional and social well-being of patients. The instrument evaluates the QoL related to eight domains: physical; emotional; function/role; social; financial; global; general symptoms and side effects such as fatigue, pain, dyspnea, appetite, sleep, nausea, constipation, diarrhea, and lung cancer specific symptoms and side effects. See Section 13.1.

**(8) Cost-Utility**

Cost-utility will be assessed on study patients using the EuroQol EQ-5D in conjunction with the OS results. See Section 13.2 for details.

# 12 STATISTICAL CONSIDERATIONS

## 12.1. Statistical Analysis

All patients who are randomized will be included in the full analysis set according to the arm into which they were randomly allocated. LC (i.e. the time to local recurrence) will be estimated using the Kaplan-Meier method for each treatment arm, and the groups will be compared using a logrank test stratified on tumour size/stage and primary tumour site, and, in addition, the Charlson comorbidity index. The hazard ratio (HR) for SBRT relative to CRT will be estimated using a Cox proportional hazard model adjusting for the strata described above.

In addition, the 3-year local recurrence will be estimated using the Kaplan-Meier method and standard errors will be estimated using Greenwood's formula. The difference in the 3-year local recurrence rates between the two arms will be reported along with 95% confidence intervals

Secondary outcomes of OS, DFS, EFS and LCSS will be analyzed in a similar fashion to LC. The secondary outcome of the proportions with RTRD in each arm will be compared using a Fisher exact test (unadjusted) and also using the Mantel-Haenszel test adjusted for tumour size/stage and primary tumour site, in addition to the Charlson comorbidity index.

Details of the QoL and Cost-utility analyses are described in Section 13 below.

Subgroups defined by tumour size/stage (T1: ≤3cm vs. T2a: >3-5cm), primary tumour site (central vs. peripheral), and Charlson comorbidity index (≤2 vs. >2) will be investigated for differential treatment effects with regard to LC. Investigation of subgroup effects for other factors determined prior to database lock will be considered as exploratory.

Acute and late toxicities will be summarized by toxicity type and grade for each treatment group.

The final analysis will occur at a median follow-up time of approximately 3 years which is consistent with reports of studies of local recurrence in lung cancer and similar to the two other randomized trials in this population (CHISEL[43], SPACE[44]) Analyses will be conducted using SAS (Cary, NC), Stata (College Station, Texas) and R (www.r-project.org).

### 12.2. Sample Size and Feasibility

The NCIC BR.25 study of 60Gy in 15 fractions recently reported 2-year LC of 88% but the curve decreases. At 3 years, LC is estimated to be 75% at best with CRT. It is expected that SBRT will result in a minimal clinically important increase of 12.5% in 3-year LC to 87.5%. In order to have 85% power to detect a difference of this magnitude (HR=0.46) with a two-sided $\alpha$=0.05 and a 2:1 (SBRT:CRT) randomization, we would require a minimum of 59 events. Assuming 3 years for patient recruitment with an additional 2 years of follow-up, we would require a total sample size of 308 patients. Although non-compliance and losses to follow-up are expected to be minimal in this cohort, we built in a 5% inflation factor. Therefore, we would need to enroll a total of 324 patients (216 SBRT and 108 CRT patients).

The radiation program at the JCC sees approximately 5000 new cancer patients per year for consultation. We recently performed a review of all stage I NSCLC patients referred for RT using a prospective ethics-approved QA database. We identified 40 patients that would potentially be eligible for the trial. Assuming some may be ineligible and only half agree to be randomized, we anticipate that at least 10 patients per year could be accrued. We have identified 5 large centres including our own (Juravinski Cancer Centre, Sunnybrook, London, Edmonton, and Winnipeg) that are expected to accrue a similar number of patients. Overall, between 16 and 20 centres across the country have agreed to participate. The majority of centres are medium to small in size with newly established or developing SBRT programs. A recent review by the one of these centres in Sudbury identified 18 stage I patients per year who would be eligible and anticipate at least 5 per year could be accrued. We expect other medium to small-sized centres to have similar numbers.

Based on these calculations, and given likely slow accrual in the first 6 months, we expect a conservative estimate of accrual of at least 110 patients per year over 3 years, meeting our target sample size. After the study opens, based on past experience, other centres would be expected to join.

## 12.3. Planned Interim Analysis

No interim analyses are planned.

# 13 HEALTH SERVICES RESEARCH

## 13.1. Quality of Life

Acute radiation therapy can cause fatigue, occasional skin irritation, pain, dyspnea, and cough secondary to radiation pneumonitis. Peak reactions are normally seen 2 weeks post radiation therapy for CRT and SBRT, although late reactions can also occur. The acute effect on QoL, as assessed by the EORTC QLQ-C30/LC-13, will be addressed at 2 weeks post radiation in both SBRT and CRT arms. SBRT involves a higher biological dose but generally treats a smaller volume of normal tissue and requires less treatment visits. Consequently, we expect SBRT to impair QoL to a lesser degree than CRT. Experts and patients suggest a difference from baseline in EORTC QLQ-C30 global health status score of 10 points or more (standard deviation=24) on a 1 to 100 scale as being an important difference to detect. With a two-sided $\alpha=0.05$ and 2:1 allocation ratio, and allowing for 5% loss, 233 patients overall (154 SBRT, 79 CRT) would provide at least 85% power to detect a difference of at least 10 points in the mean change from 2 weeks post radiation to baseline in global health status score between SBRT and CRT.

Late radiation reactions include lung fibrosis, rib fracture, and can result in severe pneumonitis or hemorrhage. Long-term QoL will be assessed at 2 years post randomization. This will give sufficient time for late effects of radiation therapy and it is expected the majority of patients will be alive at this time point. It is assumed the treatment effect at 2 years will be similar to the acute period.

The comparison of mean QoL scores at 2 weeks post-treatment and 24 months post-randomization will be performed using a linear regression model with the baseline scores as covariates, and factors defined by treatment arm, the stratification variables and the Charlson comorbidity index. As a supportive analysis, repeated measures mixed models will be used to analyze QoL profiles over time. Missing QoL data will be handled using multiple imputation methods.

## 13.2. Economic Evaluation

Healthcare costs are continuing to rise and resources for healthcare are not unlimited. SBRT is a relatively new, resource intensive modality being used to treat early stage NSCLC. There is limited data as to its effectiveness compared to more standard modalities. Based on these considerations there needs to be high quality evidence of both SBRT effectiveness and cost-utility. An economic evaluation, in the form of a cost-utility analysis, will assess the cost-effectiveness of SBRT compared to CRT in patients with lung cancer. The direct medical costs used by individual patients (e.g. hospitalizations), including the cost of administering the interventions, will be collected during the course of the study. The quantity of each resource item will be multiplied by the unit cost for that item to calculate the total cost per individual participating in the trial. Due to the unique characteristics of cost data (e.g., right skewed and excess zeroes) and a relatively small sample size, a generalized linear model will be used to analyze the cost data.[45] The outcome of the cost-

effectiveness analysis will be the quality-adjusted life year (QALY). The QALY is a preference-based measure of health outcome that combines length of life and health-related quality of life.[46] To estimate the QALY, the patient's responses to the EQ-5D questionnaire (a generic health-related quality of life questionnaire with pre-existing preference weights that can be attached to each health state), will be combined with the preference weights (or utility scores) and the survival data from the trial to generate QALY profiles. The EQ-5D will be used to estimate utility values at each assessment point. They will then be aggregated using area under the curve methodology controlling for baseline utility value to allow estimation of QALYs. The results of the cost-utility analysis will be estimated by the following equation:

$$\text{Cost-Utility} = \frac{\text{Average cost of SBRT - Average cost of CRT}}{\text{Average total QALYs for SBRT - Average QALYs for CRT}}$$

The primary perspective of the analysis will be the costs borne by the Ministries of Health. The study will be designed to measure the long-term effects of treatment on the healthcare system costs, overall survival and patient's QoL. Survival and QoL will be synthesized into quality-adjusted life-years (QALYs) through the estimation of patient's utility values at different stages of disease duration. Costs associated with lung cancer will be assumed to be in the short-term a function of treatment and in the long-term a function of disease progression and any late morbidity or comorbidity. Short-term costs and utilities will be measured by primary data collection concurrent with the clinical trial for study patients. Clinical trial data will provide length of survival for each patient. Costs and effects of disease recurrence and long-term morbidity will be obtained from the primary data collection in the clinical trial and using the Ontario case Costing Initiative Database. Cost-utility will be assessed by determining if any incremental costs associated with SBRT relative to CRT are justified given any observed improvements in QALYs.

The primary analysis will have a time horizon of 3 years, reflective of the period of primary data capture within the clinical trial. Secondary analysis will involve forecasting costs and utility values up to a time horizon of 5 years after randomization. Costs and benefits will be discounted at 5%. The uncertainty concerning the incremental cost, QALYs and cost-utility ratio will be estimated by conducting probabilistic analysis through non-parametric bootstrapping.[47] Bootstrapping allows estimation of the dispersion around an outcome of interest and the study is treated as the patient population. We will re-estimate the study sample through drawing repeated random samples of the same size as the original sample. For this study, we will obtain 5,000 estimates of costs and utility for each strategy. This approach will be used to derive 95% certainty intervals around the difference in costs, QALYs, and the incremental cost per QALY.[48] The results will be presented using cost-effectiveness acceptability curves.

## 14 STUDY SIGNIFICANCE

Radiotherapy for stage I non-small cell lung cancer is the preferred treatment option in patients medically unfit to undergo surgical resection. Radiotherapy improves local control compared to no treatment, yet long-term survival is still difficult to achieve. CRT remains the standard of care in many countries, including Canada, where access to new technologies is not widespread. In order to improve survival, and reduce cancer burden in this population, newer techniques are necessary to develop and evaluate.

SBRT represents a major advancement in radiation oncology practice, and non-randomized comparisons of SBRT versus CRT have been encouraging in terms of improving local cancer control, and potentially long-term survival, but they are not definitive. In rare instances SBRT has been associated with severe life-threatening toxicity when central lung cancers are not treated appropriately. In Canada, the technological demands and advanced expertise required to develop and maintain SBRT programs have led to some reluctance in adopting the therapy nation-wide with clear evidence of effectiveness. In the context of a public health care system, such demands are important for higher level decision making as to whether a major technological innovation should be implemented into regular clinical practice.

The highest level evidence to support a new therapy is to conduct a large phase III randomized study, adequately powered to detect improvements in important patient outcomes. A randomized study of SBRT compared to CRT is required to demonstrate superiority of SBRT in terms of improving local control in medically inoperable early stage non-small cell lung cancer patients. A thorough analysis of the added cost of SBRT and comparative effectiveness in terms of survival benefit and quality of life will determine whether the added cost of SBRT justifies widespread adoption. Such a study, if positive, would facilitate development of guidelines on the appropriate delivery of SBRT and would improve access across the country to high-quality, high-precision radiation therapy.

The variability in the use of SBRT for the treatment of NSCLC across Canada and globally is substantial. By performing a randomized study, this will enable centres across Canada to align communities of practice, and speak a common language as to the indications and appropriate and safe delivery of SBRT. A positive study will act to reduce the overall cancer burden in general, especially in a population where morbidity and mortality is high.

## 15 ETHICAL AND REGULATORY STANDARDS

This clinical trial will be conducted in accordance with the recommendations guiding physicians in biomedical research involving human patients adopted by the 18$^{th}$ World Medical Assembly, Helsinki, Finland 1964 and later revisions or the laws and regulations of the country, whichever provide the greater protection for the study participant.

This clinical trial will be conducted in compliance with the ICH guidelines for Good Clinical Practice and will adhere to national laws and regulations of the country in which the study is performed.

Personnel involved in conducting this clinical trial will be qualified by education, training and experience to perform their respective tasks.

## 15.1. Informed Consent

OCOG, as Sponsor, will provide each clinical centre with a sample Informed Consent Form (ICF). The ICF used by the local Investigator or designate for obtaining the patient's informed consent must be reviewed and approved by OCOG prior to submission to the appropriate Research Ethics Board (REB) for approval/favourable opinion.

It is the responsibility of the local Investigator or a person designated by the local Investigator and under the Investigator's responsibility, to provide each potential study patient, prior to inclusion in the study, full and adequate verbal and written information regarding the objectives and procedures of the study and the possible risks involved. The patient must be informed about their right to withdraw from the study at any time. The patient must be allowed adequate time to make an informed decision.

Prior to a patient's participation in the study, the locally approved written ICF must be signed, name filled in and personally dated by the patient or by the patient's legally acceptable representative, and by the person who conducted the informed consent discussion. A copy of the signed and dated written consent form document and any other written information should be provided to the patient.

Written informed consent will be obtained from all potentially eligible patients prior to commencing any study related procedures. Until the patient has been completely informed of the clinical trial, has freely consented to take part in the study and has signed and dated an ICF that has received documented approval by a licensed REB, no study related procedures can be performed.

## 15.2. Research Ethics Board (REB)

Prior to study commencement, the Investigator must submit this clinical trial protocol, the ICF document, recruitment materials/process, patient questionnaires, and any other written information to be provided to study patients to the appropriate REB and is required to forward to OCOG a copy of the written and dated approval/favourable opinion signed by the Chairman with REB composition.

The clinical trial (protocol number, clinical trial protocol title, version number and version date), the documents reviewed (e.g. clinical trial protocol, ICF) and the date of review should be clearly stated on the written REB approval/favourable opinion.

During the clinical trial, any amendments or modification to the study protocol or ICF document, as issued by OCOG, must be submitted to and approved by the local REB. The REB should also be informed of any event likely to affect the safety of patients or the continued conduct of the study.

Annual re-approval is required for as long as the study is open to patient accrual, study participants are being followed and until the data collection and sponsor close-out is completed.

The REB must be informed when the study is closed or has been suspended.

## 16 RESPONSIBILITIES of the INVESTIGATOR

One Qualified Investigator (QI) will oversee the trial at each clinical centre. The QI undertakes to perform the study in accordance with this clinical trial protocol, ICH guidelines for Good Clinical Practice and the applicable national regulations and local REB requirements.

The QI may appoint other individuals as he/she deems appropriate to assist in the conduct of the study. All appointed designates will be listed and provided to OCOG (Sponsor). The appointed designates will be supervised by and under the responsibility of the QI.

For the purpose of ensuring compliance with the clinical trial protocol, ICH GCP guidelines and applicable regulatory requirements, the QI agrees to permit study monitoring/auditing by or on behalf of OCOG, and inspection by applicable regulatory authorities. The Investigator agrees to allow the auditors/inspectors to have direct access to his/her study records, including source data/documents.

## 17 STUDY MONITORING and DATA HANDLING

### 17.1. Data Collection Method

Data collection is the responsibility of the designated clinical trial staff at the clinical centre under the supervision of the local QI. During the study, the QI must maintain complete and accurate documentation for the study. Study Case Report Forms (CRFs) are designed to record the disease status, treatment, intervention, all observations, follow-up and other pertinent data on each enrolled study participant. Data reported on the CRF that are derived from source documents must be consistent with the source documents. All source documents and laboratory reports must be reviewed by the designated clinical trial staff at the participating clinical centre. Adverse events must be graded, assessed for causality and reviewed by the local QI or designate.

Clinical centres will use Electronic Data Capture (EDC) to submit study data to OCOG. Electronic CRFs will be prepared for data collection requirements except for fields specific to SAEs and patient questionnaires, which will be reported on paper forms. Subjects are to be identified by subject study number, initials and date of birth, where allowable. When paper CRFs are utilized, data recorded must be neat and legible, ensuring accurate interpretation of data. Paper CRFs must be completed in ink.

The completed CRF must be reviewed promptly, signed and dated. For EDC, review and approval/signature is completed electronically through an EDC tool.

Once the submitted paper CRFs or electronic study data are received at OCOG, data verification may result in additional requests to clarify or correct the data. Data queries are tracked and archived electronically.

### 17.2. Source Document Requirements

According to the ICH guidelines for Good Clinical Practice, each participating clinical centre will maintain appropriate medical and research records for this trial. Source data are original records of clinical findings, observations, or other activities in a clinical trial necessary for the reconstruction and evaluation of the trial. The data management team must

check the CRF entries against the source documents, except for the pre-identified source data directly recorded in the CRF. The OCOG CMC will complete CRF verification by obtaining and reviewing local source documents. The ICF will include a statement by which the patient allows the Sponsor's authorized personnel, the REB, and the regulatory authorities to have direct access to source data which support the data on the CRFs. Such personnel must maintain confidentiality according to privacy legislation. It is the QI's responsibility to ensure source documentation submitted to OCOG have been de-identified and labeled with study specific identifiers (i.e., study acronym, study subject ID number and subject initials).

### 17.3. Retention of Study Records

OCOG, as sponsor, is responsible for the retention of records as per Health Canada regulations and ICH Good Clinical Practice guidelines. The QI must also maintain confidential study documentation and ensure the retention of these study documents as per national regulations and guidelines. The QI must notify OCOG (sponsor) prior to destroying any essential documents following the completion or discontinuation of the clinical trial. If the QI can no longer ensure retention of the study documentation, the QI is required to inform OCOG (sponsor) to arrange the transfer of the relevant records to a mutually agreed upon designee.

OCOG will be responsible for informing the local QI as to when trial records and documents no longer need to be retained.

## 18 CONFIDENTIALITY

All information disclosed or provided by OCOG, or produced during the clinical trial, including, but not limited to, the clinical trial protocol, the CRFs, operations manuals and the results obtained during the course of the clinical trial, is confidential. The QI at each participating centre and any person under his/her authority agrees to undertake to keep confidential and not to disclose the information to any third party without the prior written approval of OCOG. This excludes the required REB submission.

## 19 CLINICAL TRIAL PROTOCOL AMENDMENTS

Investigators should not implement any deviation from, or changes to the clinical trial protocol without written authorization from OCOG (sponsor), prior review and documented written approval from their local REB, unless the safety of the study subject is in jeopardy.

An amendment may require a change to the ICF. The Investigator must receive REB approval/favourable opinion of the revised ICF prior to the implementation of the change.

## 20 STUDY ORGANIZATION

### 20.1. Steering Committee

The Steering Committee is responsible for the overall conduct of the trial, including the design, execution, analyses, and reporting. In addition, the Steering Committee is also responsible for the assignment of responsibilities to other study committees. The Steering Committee will hold the primary responsibility for publication of the study results. This

Committee will convene on a regular basis by teleconference or face to face meetings at least every six months to address policy issues, to monitor study progress, execution and management and to review the reports from the DSMC. A list of the Steering Committee members is maintained by OCOG.

## 20.2. Data Safety Monitoring Committee

The independent Data Safety Monitoring Committee (DSMC) will include an experienced trialist, an independent statistician and a content expert. Members of the DSMC will review accumulating safety data, provided to them through the OCOG CMC, at least every six months beginning one year after study commencement. The DSMC will also review the results of the single planned interim analysis (see Section 12.3) and make recommendations to the Steering Committee regarding the discontinuation of the study in the event of unacceptable risk or extreme benefit.

## 20.3. Study Coordination

The OCOG CMC, located at the Juravinski Hospital in Hamilton, Ontario, is responsible for the overall study management including finance and contracts, implementation of the study protocol logistics, patient allocation, data management, quality assurance and statistical analysis. The CMC is responsible for monitoring study execution, particularly with regard to methodological aspects and ensuring each clinical centre adheres to the study protocol. Web-based registration or randomization will be performed by the clinical centres utilizing the CMC's Interactive Registration/Randomization System (IRIS). A secure and confidential electronic study database is maintained by the CMC. Data collection will be performed via an EDC system. The CMC's Online Remote Collection of Clinical Information and Data (ORCCID) system incorporates a clinical database, data query process, and visit management tracking to ensure data is complete, accurate, of high quality and is reported or submitted according to required timelines. Designated personnel at participating clinical centres will be provided with member access to OCOG's website to obtain study documents, operations manuals, monthly status reports and newsletters.

An in-house Ethics and Regulatory Affairs Officer will complete and submit the required provincial application to OCREB for Ontario centres, and facilitate the local REB applications; ensuring required start-up documentation is obtained from each clinical centre prior to centre activation.

The CMC will keep the Steering Committee informed of the study progress and report any problems or issues throughout the course of the study. In addition, the CMC at OCOG will prepare monthly status reports as well as summary information and reports for the various study committees and will provide methodological and administrative support to all study committees, Investigators and other study personnel.

## 20.4. Central Adjudication Committee

A Central Adjudication Committee comprised of independent experts in the areas of radiation oncology, SBRT, radiology, and hematology will be responsible for assessing recurrences and deaths (including RTRDs). The CMC at OCOG will provide support to the Central Adjudication Committee and establish a process for the collection, review and

documentation of the adjudication items. The members of the Central Adjudication Committee who perform adjudication will not be involved in the treatment of study patients.

## 21 SCIENTIFIC REPORTING and PUBLICATION

This clinical trial protocol was developed by the Principal Investigator(s) and study Steering Committee, with the assistance of OCOG.

The Steering Committee is responsible for the scientific reporting, publishing and presentation of the study results. Authorship will be determined by the Steering Committee and will be guided by the extent of participation in the development of the protocol, accrual of patients to the study, involvement in the study analysis and the drafting of the final manuscript. Results of the study will be disseminated through publications and presentations at international meetings. Any other publication or presentation related to the study and the results by any investigator or participant must receive prior approval from the Steering Committee. No other publication or presentation is permitted before the primary publication or presentation by the Steering Committee.

The information developed during the conduct of this clinical study is considered confidential.

## 22 REFERENCES


1 Ginsberg RJ, Rubinstein LV. Randomized trial of lobectomy versus limited resection for T1 N0 non-small cell lung cancer. Lung Cancer Study Group. Ann Thorac Surg 1995; 60(3):615-622; Discussion 622-613

2 Nesbitt JC, Putnam JB, Jr., Walsh GL, Roth JA, Mountain CF. Survival in early-stage non-small cell lung cancer. Ann Thorac Surg 1995; 60(2):466-472.

3 Wisnivesky JP, Bonomi M, Henschke C, Iannuzzi M, McGinn T. Radiation therapy for the treatment of unresected stage I-II non-small cell lung cancer. Chest 2005; 128(3):1461-1467.

4 Bach PB, Cramer LD, Warren JL, Begg CB. Racial differences in the treatment of early-stage lung cancer. N Engl J Med 1999; 341(16):1198-1205.

5 Qiao X, Tullgren O, Lax I, Sirzen F, Lewensohn R. The role of radiotherapy in treatment of stage I non-small cell lung cancer. Lung Cancer 2003; 41(1):1-11.

6 Zimmermann FB, Bamberg M, Molls M, Jeremic B. Radiation therapy alone in early stage non-small cell lung cancer. Semin Surg Oncol 2003; 21(2):91-97.

7 Rowell NP, Williams CJ. Radical radiotherapy for stage I/II non-small cell lung cancer in patients not sufficiently fit for or declining surgery (medically inoperable): a systematic review. Thorax 2001; 56(8):628-638

8 Sibley GS. Radiotherapy for patients with medically inoperable Stage I non-small cell lung carcinoma: smaller volumes and higher doses - a review. Cancer 1998; 82(3):433-438.

9 Bogart JA, Hodgson L, Seagren SL, Blackstock AW, Wang X, Lenox R, Turrisi AT, 3rd, Reilly J, Gajra A, Vokes EE, Green MR. Phase I study of accelerated conformal radiotherapy for stage I non-small cell lung cancer in patients with pulmonary dysfunction: CALGB 39904. J Clin Oncol 2010; 28(2):202-206.

10 Slotman BJ, Antonisse IE, Njo KH. Limited field irradiation in early stage (T1-2N0) non-small cell lung cancer. Radiother Oncol 1996; 41(1):41-44.

11 Soliman H, Cheung P, Yeung L, Poon I, Balogh J, Barbera L, Spayne J, Danjoux C, Dahele M, Ung Y. Accelerated hypofractionated radiotherapy for early-stage non-small-cell lung cancer: long-term results. Int J Radiat Oncol Biol Phys 2011; 79(2):459-465.

12 Potters L, Kavanagh B, Galvin JM, Hevezi JM, Janjan NA, Larson DA, Mehta MP, Ryu S, Steinberg M, Timmerman R, Welsh JS, Rosenthal SA. American Society for Therapeutic Radiology and Oncology (ASTRO) and American College of Radiology (ACR) practice guideline for the performance of stereotactic body radiation therapy. Int J Radiat Oncol Biol Phys 2010; 76(2):326-332.

13 Hadziahmetovic M, Loo BW, Timmerman RD, Mayr NA, Wang JZ, Huang Z, Grecula JC, Lo SS. Stereotactic body radiation therapy (stereotactic ablative radiotherapy) for stage I non-small cell lung cancer updates of radiobiology, techniques, and clinical outcomes. Discov Med 2010; 9(48):411-417.

14 Baumann P, Nyman J, Hoyer M, Gagliardi G, Lax I, Wennberg B, Drugge N, Ekberg L, Friesland S, Johansson KA, Lund JS, Morhed E, Nilsson K, Levin N, Paludan M, Sederholm C, Traberg A, Wittgren L, Lewensohn R. Stereotactic body radiotherapy for medically inoperable patients with stage I non-small cell lung cancer - a first report of toxicity related to COPD/CVD in a non-randomized prospective phase II study. Radiother Oncol 2008; 88(3):359-367.

15 Fakiris AJ, McGarry RC, Yiannoutsos CT, Papiez L, Williams M, Henderson MA, Timmerman R. Stereotactic body radiation therapy for early-stage non-small-cell lung carcinoma: four-year results of a prospective phase II study. Int J Radiat Oncol Biol Phys 2009; 75(3):677-682.

16 Koto M, Takai Y, Ogawa Y, Matsushita H, Takeda K, Takahashi C, Britton KR, Jingu K, Takai K, Mitsuya M, Nemoto K, Yamada S. A phase II study on stereotactic body radiotherapy for stage I non-small cell lung cancer. Radiother Oncol 2007; 85(3):429-434.

17 Nagata Y, Takayama K, Matsuo Y, Norihisa Y, Mizowaki T, Sakamoto T, Sakamoto M, Mitsumori M, Shibuya K, Araki N, Yano S, Hiraoka M. Clinical outcomes of a phase I/II study of 48Gy of stereotactic body radiotherapy in 4 fractions for primary lung cancer using a stereotactic body frame. Int J Radiat Oncol Biol Phys 2005; 63(5):1427-1431.

18 Timmerman R, Paulus R, Galvin J, Michalski J, Straube W, Bradley J, Fakiris A, Bezjak A, Videtic G, Johnstone D, Fowler J, Gore E, Choy H. Stereotactic body radiation therapy for inoperable early stage lung cancer. JAMA 2010; 303(11):1070-1076.

19 Videtic GM, Stephans K, Reddy C, Gajdos S, Kolar M, Clouser E, Djemil T. Intensity-modulated radiotherapy-based stereotactic body radiotherapy for medically inoperable early-stage lung cancer: excellent local control. Int J Radiat Oncol Biol Phys 2010; 77(2):344-349.

20 Taremi M, Hope A, Dahele M, Pearson S, Fung S, Purdie T, Brade A, Cho J, Sun A, Bissonnette JP, Bezjak A. Stereotactic body radiotherapy for medically inoperable lung cancer: prospective, single-center study of 108 consecutive patients. Int J Radiat Oncol Biol Phys 2012; 82(2):967-973.

21 Timmerman R, McGarry R, Yiannoutsos C, Papiez L, Tudor K, DeLuca J, Ewing M, Abdulrahman R, DesRosiers C, Williams M, Fletcher J. Excessive toxicity when treating central tumours in a phase II study of stereotactic body radiation therapy for medically inoperable early-stage lung cancer. J Clin Oncol 2006; 24(30):4833-4839.

22 Andrews DW, Scott CB, Sperduto PW, Flanders AE, Gaspar LE, Schell MC, Werner-Wasik M, Demas W, Ryu J, Bahary JP, Souhami L, Rotman M, Mehta MP, Curran WJ, Jr. Whole brain radiation therapy with or without stereotactic radiosurgery boost for patients with one to three brain metastases: phase III results of the RTOG 9508 randomised trial. Lancet 2004; 363(9422):1665-1672.

23 Aoyama H, Shirato H, Tago M, Nakagawa K, Toyoda T, Hatano K, Kenjyo M, Oya N, Hirota S, Shioura H, Kunieda E, Inomata T, Hayakawa K, Katoh N, Kobashi G. Stereotactic radiosurgery plus whole-brain radiation therapy vs stereotactic radiosurgery alone for treatment of brain metastases: a randomized controlled trial. JAMA 2006; 295(21):2483-2491.

24 Maziak DE, Darling GE, Inculet RI, Gulenchyn KY, Driedger AA, Ung YC, Miller JD, Gu CS, Cline KJ, Evans WK, Levine MN. Positron emission tomography in staging early lung cancer: a randomized trial. Ann Intern Med 2009; 151(4):221-228, W-248.

25 Fowler JF. The linear-quadratic formula and progress in fractionated radiotherapy. Br J Radiol 1989; 62(740):679-694.

26 Faria SL, Souhami L, Portelance L, Duclos M, Vuong T, Small D, Freeman CR. Absence of toxicity with hypofractionated 3-dimensional radiation therapy for inoperable, early stage non-small cell lung cancer. Radiat Oncol 2006; 1:42.

27 Cheung P. A Phase II Study of Accelerated Hypofractionated 3-Dimensional Conformal Radiotherapy (3DCRT) For Inoperable Stage I/II Non-Small Cell Lung Cancer (NSCLC). http://www.clinicaltrials.gov/ct2/show/NCT00346320. 2012.

28 Cho BC, Bezjak A, Dawson LA. Image guidance in non-small cell lung cancer. Semin Radiat Oncol 2010; 20(3):164-170.

29 Brock J, Bedford J, Partridge M, McDonald F, Ashley S, McNair HA, Brada M. Optimising stereotactic body radiotherapy for non-small cell lung cancer with volumetric intensity-modulated arc therapy--a planning study. Clin Oncol (R Coll Radiol) 2012; 24(1):68-75.

30 Jaffray D, Kupelian P, Djemil T, Macklis RM. Review of image-guided radiation therapy. Expert Rev Anticancer Ther 2007; 7(1):89-103.

31 Zhang J, Yang F, Li B, Li H, Liu J, Huang W, Wang D, Yi Y, Wang J. Which is the optimal biologically effective dose of stereotactic body radiotherapy for Stage I non-small-cell lung cancer? A meta-analysis. Int J Radiat Oncol Biol Phys 2011; 81(4):e305-316.

32 Grutters JP, Kessels AG, Pijls-Johannesma M, De Ruysscher D, Joore MA, Lambin P. Comparison of the effectiveness of radiotherapy with photons, protons and carbon-ions for non-small cell lung cancer: a meta-analysis. Radiother Oncol 2010; 95(1):32-40.

33 Lagerwaard FJ, Haasbeek CJ, Smit EF, Slotman BJ, Senan S. Outcomes of risk-adapted fractionated stereotactic radiotherapy for stage I non-small-cell lung cancer. Int J Radiat Oncol Biol Phys 2008; 70(3):685-692.

34 Haasbeek CJ, Lagerwaard FJ, Slotman BJ, Senan S. Outcomes of stereotactic ablative radiotherapy for centrally located early-stage lung cancer. J Thorac Oncol 2011; 6(12):2036-2043.

35 Mitera G, Swaminath A, Rudoler D, Seereeram C, Giuliani M, Leighl N, Gutierrez E, Dobrow M, Coyte P, Yung T, Bezjak A, Hope A. Cost-Effectiveness Analysis Comparing Conventional Versus Stereotactic Body Radiotherapy For Surgically Ineligible Stage I Non-Small Cell Lung Cancer. Journal of Thoracic Oncology 2012; 6(Suppl 2):S280.

36 Pan H, Simpson DR, Mell LK, Mundt AJ, Lawson JD. A survey of stereotactic body radiotherapy use in the United States. Cancer 2011; 117(19):4566-4572.

37 Lund C, Halperin R, Schellenberg D. Stereotactic body radiotherapy (SBRT). A national survey of Canadian radiotherapy centres not currently treating patients with SBRT. Radiother Oncol 2011; 100(S1):8.

38 Ball D. A Randomised Phase III Trial of Highly Conformal Hypofractionated Image Guided ("Stereotactic") Radiotherapy (HypoRT) Versus Conventionally Fractionated Radiotherapy (ConRT) for Inoperable Early Stage I Non-small Cell Lung Cancer (CHISEL).http://www.clinicaltrials.gov/ct2/show/NCT01014130. 2012.

39 Whelan TJ, Olivotto I, Ackerman I, Chapman JW, Chua B, Nabid A, Vallis KA, White JR, Rousseau P, Fortin A, Pierce LJ, Manchul L, Craighead P, Nolan MC, Bowen J, McCready DR, Pritchard KI, Levine M. NCIC-CTG MA.20: An intergroup trial of regional nodal irradiation in early breast cancer. J Clin Oncol 2011; S1:LBA1003.

40 Maringwa JT, Quinten C, King M, Ringash J, Osoba D, Coens C, Martinelli F, Vercauteren J, Cleeland CS, Flechtner H, Gotay C, Greimel E, Taphoorn MJ, Reeve BB, Koch JS, Weis J, Smit EF, van Meerbeeck JB, Bottomley A. Minimal important differences for interpreting health-related quality of life scores from the EORTC QLQ-C30 in lung cancer patients participating in randomized controlled clinical trials. Support Care Cancer 2011; 10(11):1753-1760.

41 Gridelli C, Perrone F, Nelli F, Ramponi S, De Marinis F. Quality of life in lung cancer patients. Ann Oncol 2001; 12 Suppl 3:S21-25.

42 Huang K, Dahele M, Senan S, Guckenberger M, Rodrigues GB, Ward A, Boldt RG, Palma DA. Radiographic changes after lung stereotactic ablative radiotherapy (SABR) - can we distinguish recurrence from fibrosis? A systematic review of the literature. Radiother Oncol 2012; 102(3):335-342.

43 Ball D, Mai GT, Vinod S, et al. Stereotactic ablative radiotherapy versus standard radiotherapy in stage 1 non-small-cell lung cancer (TROG 09.02 CHISEL): a phase 3, open-label, randomised controlled trial. Lancet Oncol. 2019; 20(4):494-503

44 Nyman J, Hallqvist A, Lund JÅ, Brustugun OT, Bergman B, Bergström P, Friesland S, Lewensohn R, Holmberg E, Lax I. SPACE - A randomized study of SBRT vs conventional fractionated radiotherapy in medically inoperable stage I NSCLC. Radiother Oncol. 2016 Oct;121(1):1-8. doi: 10.1016/j.radonc.2016.08.015. Epub 2016 Sep 3. PMID: 27600155.

45 Mihaylova B, Briggs A, O'Hagan A, Thompson SG. Review of statistical methods for analyzing healthcare resources and costs. Health Econ 2011; 20(8):897-916.

46 Torrance GW, Feeny D. Utilities and quality-adjusted life years. Int J Technol Assess Health Care 1989;5(4):559-75.

47 Campbell MK, Torgerson DJ. Bootstrapping: estimating confidence intervals for cost-effectiveness ratios. QJM 1999; 92(3):177-182.

48 Chaudhary MA, Stearns SC. Estimating confidence intervals for cost-effectiveness ratios: an example from a randomized trial. Stat Med1996; 15(13):1447-1458.

49 Oken MM, Creech RH, Tormey DC, Horton J, Davis TE, McFadden ET, Carbone PP: Toxicity and Response Criteria Of The Eastern Cooperative Oncology Group. Am J Clin Oncol 1982; 5:649-655.

# Appendix I: Schedule of Study Assessments and Evaluations

| Assessments/Tests | Baseline Prior to Randomization | | Follow-up Assessments | | | | |
|---|---|---|---|---|---|---|---|
| | | | | Post-Randomization | | | |
| | | | 2 Weeks Post RT | 3 Months | 6, 9, 12, 18, 24 Months | 30 Months, 3 Years | 4, 5 Years |
| Informed Consent | X | RADIATION TREATMENT | | | | | |
| Demographics | X | | | | | | |
| ECOG Performance Status | X | | | | | | |
| PFTs | X | | | | | | |
| Comorbidity Status | X | | | | | | |
| Physical Exam: | | | | | | | |
| • Height and weight | X | | | | | | |
| TNM Staging† with: | | | | | | | |
| • CT thorax | X | | | | | | |
| • Full Body FDG PET-CT | X | | | | | | |
| • CT/MRI Brain (if performed) | X | | | | | | |
| QoL: | | | | | | | |
| • EORTC QLQ-C30 | X | | X | X | X | | |
| • EORTC QLQ-LC13 | X | | X | X | X | | |
| Resource Utilization: | | | | | | | |
| • EQ-5D | X | | X | X | X | X | |
| • Doctor visits, ER visits, Hospitalizations | | | | | X‡ | | |
| Toxicity (NCI CTCAE): | | | | | | | |
| • Symptom assessment | X | | | | | | |
| • Acute | | | X | X | | | |
| • Late | | | | | X | X | X |
| Cancer Recurrence Testing* | | | | | | | |
| • CT thorax | | | | X | X | X | X |
| Survival | | | X | X | X | X | X |

† FDG PET-CT must be performed within 3 months of randomization (then a CT thorax within 8 weeks is not necessary). If PET-CT is not performed within 3 months of randomization then an up-to-date CT within 8 weeks is acceptable. There must be no change in clinical stage. Brain imaging investigations, performed within 3 months prior to randomization, are preferred but not required.

* At the time of first recurrence, subjects will be fully staged with a clinical exam, CT abdomen/thorax, CT/MRI brain and/or FDG PET-CT scan.

‡ Health Resource Utilization CRF used to capture doctor/hospital visits at 6, 12, 18 and 24 months for all patients in control arm and half of patients in SBRT arm.

# Appendix II: TNM Cancer Staging
## AJCC Lung 7th Edition 2009

### Primary Tumour (T)

**TX** Primary tumour cannot be assessed, or tumour proven by the presence of malignant cells in sputum or bronchial washings but not visualized by imaging or bronchoscopy
**T0** No evidence of primary tumour
**Tis** Carcinoma in situ
**T1** Tumour 3 cm or less in greatest dimension, surrounded by lung or visceral pleura, without bronchoscopic evidence of invasion more proximal than the lobar bronchus (for example, not in the main bronchus)*
**T1a** Tumour 2 cm or less in greatest dimension
**T1b** Tumour more than 2 cm but 3 cm or less in greatest dimension
**T2** Tumour more than 3 cm but 7 cm or less or tumour with any of the following features (T2 tumours with these features are classified T2a if 5 cm or less): involves main bronchus, 2 cm or more distal to the carina; invades visceral pleura (PL1 or PL2); associated with atelectasis or obstructive pneumonitis that extends to the hilar region but does not involve the entire lung
**T2a** Tumour more than 3 cm but 5 cm or less in greatest dimension
**T2b** Tumour more than 5 cm but 7 cm or less in greatest dimension
**T3** Tumour more than 7 cm or one that directly invades any of the following: parietal pleural (PL3), chest wall (including superior sulcus tumours), diaphragm, phrenic nerve, mediastinal pleura, parietal pericardium; or tumour in the main bronchus less than 2 cm distal to the carina* but without involvement of the carina; or associated atelectasis or obstructive pneumonitis of the entire lung or separate tumour nodule(s) in the same lobe
**T4** Tumour of any size that invades any of the following: mediastinum, heart, great vessels, trachea, recurrent laryngeal nerve, esophagus, vertebral body, carina, separate tumour nodule(s) in a different ipsilateral lobe

### Regional Lymph Nodes (N)

**NX** Regional lymph nodes cannot be assessed
**N0** No regional lymph node metastases
**N1** Metastasis in ipsilateral peribronchial and/or ipsilateral hilar lymph nodes and intrapulmonary nodes, including involvement by direct extension
**N2** Metastasis in ipsilateral mediastinal and/or subcarinal lymph node(s)
**N3** Metastasis in contralateral mediastinal, contralateral hilar, ipsilateral or contralateral scalene, or supraclavicular lymph node(s)

### Distant Metastasis (M)

**M0** No distant metastasis
**M1** Distant metastasis
**M1a** Separate tumour nodule(s) in a contralateral lobe, tumour with pleural nodules or malignant pleural (or pericardial) effusion**
**M1b** Distant metastasis (in extrathoracic organs)

## Anatomic Stage/Prognostic Groups

**Occult Carcinoma** TX N0 M0

**Stage 0** Tis N0 M0

| | |
|---|---|
| **Stage IA** | T1a N0 M0 |
| | T1b N0 M0 |
| **Stage IB** | T2a N0 M0 |
| **Stage IIA** | T2b N0 M0 |
| | T1a N1 M0 |
| | T1b N1 M0 |
| | T2a N1 M0 |
| **Stage IIB** | T2b N1 M0 |
| | T3 N0 M0 |
| **Stage IIIA** | T1a N2 M0 |
| | T1b N2 M0 |
| | T2a N2 M0 |
| | T2b N2 M0 |
| | T3 N1 M0 |
| | T3 N2 M0 |
| | T4 N0 M0 |
| | T4 N1 M0 |
| **Stage IIIB** | T1a N3 M0 |
| | T1b N3 M0 |
| | T2a N3 M0 |
| | T2b N3 M0 |
| | T3 N3 M0 |
| | T4 N2 M0 |
| | T4 N3 M0 |
| **Stage IV** | Any T Any N M1a |
| | Any T Any N M1b |

### Notes

* The uncommon superficial spreading tumour of any size with its invasive component limited to the bronchial wall, which may extend proximally to the main bronchus, is also classified as T1a.

** Most pleural (and pericardial) effusions with lung cancer are due to tumour. In a few patients, however, multiple cytopathologic examinations of pleural (pericardial) fluid are negative for tumour, and the fluid is non-bloody and is not an exudate. Where these elements and clinical judgment dictate that the effusion is not related to the tumour, the effusion should be excluded as a staging element and the patient should be classified as M0.

## Appendix III: ECOG Performance Status

| Grade | Description of Performance Status |
|---|---|
| 0 | Fully active, able to carry on all pre-disease performance without restriction |
| 1 | Restricted in physically strenuous activity but ambulatory and able to carry out work of a light or sedentary nature, e.g., light house work, office work |
| 2 | Ambulatory and capable of all self-care but unable to carry out any work activities. Up and about more than 50% of waking hours |
| 3 | Capable of only limited self-care, confined to bed or chair more than 50% of waking hours |
| 4 | Completely disabled. Cannot carry on any self-care. Totally confined to bed or chair |
| 5 | Dead |

The ECOG Performance Status is in the public domain therefore available for public use. To duplicate the scale, please cite the reference above and credit the Eastern Cooperative Oncology Group, Robert Comis MD, Group Chair.[49]

# Appendix IV: Charlson Comorbidity Index

| CATEGORY | CONDITION | WEIGHT |
|---|---|---|
| **A** | • Myocardial infarct<br>• Congestive heart failure<br>• Peripheral vascular disease<br>• Cerebrovascular disease (except hemiplegia)<br>• Dementia<br>• Chronic pulmonary disease<br>• Connective tissue disease<br>• Ulcer disease<br>• Mild liver disease<br>• Diabetes (without complications) | **1** |
| **B** | • Diabetes with end-organ damage (nephropathy, retinopathy, vasculopathy)<br>• Hemiplegia<br>• Moderate or severe renal disease<br>• $2^{nd}$ solid tumour (non-metastatic)<br>• Leukemia<br>• Lymphoma, multiple myeloma | **2** |
| **C** | • Moderate or severe liver disease | **3** |
| **D** | • $2^{nd}$ metastatic solid tumour<br>• AIDS | **6** |

**Charlson Score** = $(\boldsymbol{n_A} \times 1) + (\boldsymbol{n_B} \times 2) + (\boldsymbol{n_C} \times 3) + (\boldsymbol{n_D} \times 6)$

where $\boldsymbol{n_x}$ = number of conditions in each category ($\boldsymbol{x}$ = A, B, C and D)

## Appendix V. Quality of Life Questionnaires

# EORTC QLQ-C30 (version 3)

We are interested in some things about you and your health. Please answer all of the questions yourself by circling the number that best applies to you. There are no "right" or "wrong" answers. The information that you provide will remain strictly confidential.

Please fill in your initials: |__|__|__|

Your birthdate (Day, Month, Year): |__|__|__|__|__|__|__|__|

Today's date (Day, Month, Year): |__|__|__|__|__|__|__|__|

---

| | Not at All | A Little | Quite a Bit | Very Much |
|---|---|---|---|---|
| 1. Do you have any trouble doing strenuous activities, like carrying a heavy shopping bag or a suitcase? | 1 | 2 | 3 | 4 |
| 2. Do you have any trouble taking a long walk? | 1 | 2 | 3 | 4 |
| 3. Do you have any trouble taking a short walk outside of the house? | 1 | 2 | 3 | 4 |
| 4. Do you need to stay in bed or a chair during the day? | 1 | 2 | 3 | 4 |
| 5. Do you need help with eating, dressing, washing yourself or using the toilet? | 1 | 2 | 3 | 4 |

| **During the past week:** | Not at All | A Little | Quite a Bit | Very Much |
|---|---|---|---|---|
| 6. Were you limited in doing either your work or other daily activities? | 1 | 2 | 3 | 4 |
| 7. Were you limited in pursuing your hobbies or other leisure time activities? | 1 | 2 | 3 | 4 |
| 8. Were you short of breath? | 1 | 2 | 3 | 4 |
| 9. Have you had pain? | 1 | 2 | 3 | 4 |
| 10. Did you need to rest? | 1 | 2 | 3 | 4 |
| 11. Have you had trouble sleeping? | 1 | 2 | 3 | 4 |
| 12. Have you felt weak? | 1 | 2 | 3 | 4 |
| 13. Have you lacked appetite? | 1 | 2 | 3 | 4 |
| 14. Have you felt nauseated? | 1 | 2 | 3 | 4 |
| 15. Have you vomited? | 1 | 2 | 3 | 4 |

## During the past week:

| | Not at All | A Little | Quite a Bit | Very Much |
|---|---|---|---|---|
| 16. Have you been constipated? | **1** | **2** | **3** | **4** |
| 17. Have you had diarrhea? | **1** | **2** | **3** | **4** |
| 18. Were you tired? | **1** | **2** | **3** | **4** |
| 19. Did pain interfere with your daily activities? | **1** | **2** | **3** | **4** |
| 20. Have you had difficulty in concentrating on things, like reading a newspaper or watching television? | **1** | **2** | **3** | **4** |
| 21. Did you feel tense? | **1** | **2** | **3** | **4** |
| 22. Did you worry? | **1** | **2** | **3** | **4** |
| 23. Did you feel irritable? | **1** | **2** | **3** | **4** |
| 24. Did you feel depressed? | **1** | **2** | **3** | **4** |
| 25. Have you had difficulty remembering things? | **1** | **2** | **3** | **4** |
| 26. Has your physical condition or medical treatment interfered with your family life? | **1** | **2** | **3** | **4** |
| 27. Has your physical condition or medical treatment interfered with your social activities? | **1** | **2** | **3** | **4** |
| 28. Has your physical condition or medical treatment caused you financial difficulties? | **1** | **2** | **3** | **4** |

## For the following questions, please circle the number between 1 and 7 that best applies to you:

29. How would you rate your overall health during the past week?

| 1 | 2 | 3 | 4 | 5 | 6 | 7 |
|---|---|---|---|---|---|---|
| **Very poor** | | | | | | **Excellent** |

30. How would you rate your overall quality of life during the past week?

| 1 | 2 | 3 | 4 | 5 | 6 | 7 |
|---|---|---|---|---|---|---|
| **Very poor** | | | | | | **Excellent** |

# EORTC QLQ-LC13

Patients sometimes report that they have the following symptoms or problems. Please indicate the extent to which you have experienced these symptoms or problems **during the past week**. Please answer by circling the number that best applies to you.

| **During the past week:** | **Not at All** | **A Little** | **Quite a Bit** | **Very Much** |
|---|---|---|---|---|
| 31. How much did you cough? | 1 | 2 | 3 | 4 |
| 32. Did you cough up blood? | 1 | 2 | 3 | 4 |
| 33. Were you short of breath when you rested? | 1 | 2 | 3 | 4 |
| 34. Were you short of breath when you walked? | 1 | 2 | 3 | 4 |
| 35. Were you short of breath when you climbed stairs? | 1 | 2 | 3 | 4 |
| 36. Have you had a sore mouth or tongue? | 1 | 2 | 3 | 4 |
| 37. Have you had trouble swallowing? | 1 | 2 | 3 | 4 |
| 38. Have you had tingling hands or feet? | 1 | 2 | 3 | 4 |
| 39. Have you had hair loss? | 1 | 2 | 3 | 4 |
| 40. Have you had pain in your chest? | 1 | 2 | 3 | 4 |
| 41. Have you had pain in your arm or shoulder? | 1 | 2 | 3 | 4 |
| 42. Have you had pain in other parts of your body?<br>***If yes***, where: ____________________ | 1 | 2 | 3 | 4 |
| 43. Did you take any medicine for pain? | 1 Yes | 2 No | | |
| ***If yes***, how much did it help? | 1 | 2 | 3 | 4 |

## Appendix VI: EQ-5D Questionnaire

# EQ-5D Descriptive Health Questionnaire

*(Canadian English Version)*

By placing a check-mark in one box in each group below, please indicate which statements best describe your own state of health today.

**Mobility**

I have no problems in walking about ............................................................ ❑
I have some problems in walking about........................................................ ❑
I am confined to bed ...................................................................................... ❑

**Self-Care**

I have no problems with self-care ................................................................. ❑
I have some problems washing or dressing myself...................................... ❑
I am unable to wash or dress myself ............................................................. ❑

**Usual Activities** (*e.g. work, study, housework, family or leisure activities)*

I have no problems with performing my usual activities.............................. ❑
I have some problems with performing my usual activities ......................... ❑
I am unable to perform my usual activities................................................... ❑

**Pain/Discomfort**

I have no pain or discomfort .......................................................................... ❑
I have moderate pain or discomfort ............................................................... ❑
I have extreme pain or discomfort ................................................................. ❑

**Anxiety/Depression**

I am not anxious or depressed........................................................................ ❑
I am moderately anxious or depressed .......................................................... ❑
I am extremely anxious or depressed ............................................................. ❑

Best imaginable
state of health

# EQ-5D VAS

To help people say how good or bad their state of health is, we have drawn a scale (rather like a thermometer) on which the best state you can imagine is marked 100 and the worst state you can imagine is marked 0.

We would like you to indicate on this scale how good or bad your own health is today, in your opinion. Please do this by drawing a line from the box below to whichever point on the scale indicates how good or bad your state of health is today.

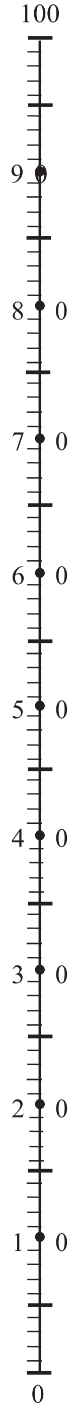


**Your own state of health today**

©1990 EuroQol Group EQ-5D™

Worst imaginable
state of health
