# Supplementary material for: Stereotactic vs Hypofractionated Radiotherapy for Inoperable Stage I Non–Small Cell Lung Cancer: The LUSTRE Phase 3 Randomized Clinical Trial
Source: JAMA Oncol. 2024 Sep 19;10(11):1571–5. doi: 10.1001/jamaoncol.2024.3089 (PMC11413752; doi:10.1001/jamaoncol.2024.3089)
Supplement: Supplement 2. — eAppendix 1. Trial Sites and Investigators eAppendix 2. Radiation Planning Guide eAppendix 3. Definition of Outcomes and Censoring Rules eAppendix 4. Baseline Characteristics eAppendix 5. Cumulative Incidence of Local Failure, Patterns of Failure, and Causes of Death eAppendix 6. Further Toxic Effects Details eAppendix 7. SBRT vs CRT RCT Dose Comparison eAppendix 8. Recruitment Table by Center [file jamaoncol-e243089-s002.pdf]

Supplemental Online Content

Swaminath A, Parpia S, Wierzbicki M, et al. Stereotactic vs hypofractionated radiotherapy for inoperable stage I non–small cell lung cancer: the LUSTRE phase 3 randomized clinical trial. *JAMA Oncol*. Published online September 19, 2024. doi:10.1001/jamaoncol.2024.3089

eAppendix 1. Trial Sites and Investigators

eAppendix 2. Radiation Planning Guide

eAppendix 3. Definition of Outcomes and Censoring Rules

eAppendix 4. Baseline Characteristics

eAppendix 5. Cumulative Incidence of Local Failure, Patterns of Failure, and Causes of Death

eAppendix 6. Further Toxic Effects Details

eAppendix 7. SBRT vs CRT RCT Dose Comparison

eAppendix 8. Recruitment Table by Center

This supplementary material has been provided by the authors to give readers additional information about their work.

© 2024 Swaminath A et al. *JAMA Oncology*.

eAppendix 1. Trial Sites and Investigators

Juravinski Cancer Centre
Site Investigator: Anand Swaminath
Co-Investigators: Gordon Okawara, Ranjan Sur, Jim Wright
Physicist: Marcin Wierzbicki

Cancer Centre of Southeastern Ontario at Kingston
Site Investigator: Timothy E. Owen
Co-Investigators: Allison Ashworth, Conrad Falkson, Aamer Mahmud, Sou Thain
Physicist: Chandra Joshi

Niagara Health System, St. Catharines
Site Investigator: Theos Tsakiridis
Co-Investigators: Gordon Okawara
Physicist: Ranjini Tolakanahalli

London Regional Cancer Centre
Site Investigator: Brian Yaremko
Co-Investigators: Alexander Louie, David Palma, George Rodrigues, Edward Yu
Physicist: Stewart Gaede

Thunder Bay Regional Health Sciences Centre
Site Investigator: Kevin Ramchandar
Co-Investigators: Margaret Anthes
Physicist: Rita Murphy

Windsor Regional Cancer Centre
Site Investigator: Khalid Hirmiz
Co-Investigators: Ming Pan, Junaid Yousuf
Physicist: John Agapito

BC Cancer -Vancouver Island Centre
Site Investigator: Elaine Sze-Sze Wai
Co-Investigators: Tanya Berrang, Paul Blood, Jean-Marc Bourque, Juliana Caon, Saibishkumar Parameswaran, Sally Smith, Susan Tyler
Physicist: Parminder Basran

CancerCare Manitoba
Site Investigator: Naseer Ahmed
Co-Investigators: Bashir Bashir, Amitava Chowdhury, Arbind Dubey, Julian Kim, Ahmet Leylek
Physicist: Sankar Venkataraman

Tom Baker Cancer Centre
Site Investigator: Harold Lau
Co-Investigators: Steve J. Angyalfi, Elizabeth Kurien, Gerald Lim

© 2024 Swaminath A et al. *JAMA Oncology*.

Physicist: Nicolas Ploquin

Cross Cancer Institute
Site Investigator: Zsolt Gabos
Co-Investigators: Brock Debenham, Alysa M. Fairchild, Tirath Nijjar, Wilson Roa, Diane Marie Severin, Don Yee
Physicist: Heather Warkentin

Horizon Health Network -Saint John Regional Hospital
Site Investigator: Robert Thompson
Co-Investigators: Amanda Caissie, Holly Campbell, Elisa Chan, Farah Naz
Physicist: Jon Dysart, Edward Lawrence

McGill University Health Centre (MUHC) -Glen Site
Site Investigator: Sergio Faria
Co-Investigators: Bassam AbdulKarim, Marie Duclos, Neil Kopek
Physicist: Russell Ruo

Hopital Maisonneuve -Rosemont
Site Investigator: Alexis Bujold
Co-Investigators: Christian Bou Karam, Pierre Chabot, Bernard Fortin, Benoit Laliberte, David Nguyen, Alma Sylvestre
Physicist: Dominique Martin

CHUM -Centre Hospitalier de L'Université de Montréal
Site Investigator: Edith Filion
Co-Investigators: Houda Bahig, Marie-Pierre Campeau, Toni Vu
Physicist: Dominic Beliveau Nadeau

Allan Blair Cancer Centre
Site Investigator: Nelson Leong
Co-Investigators: Asim Amjad, Gillian Bailey, Michelle Ferguson, Joshua Giambattista, Elaine Liu, Shazia Mahmood, Dilip Panjwani, Evgeny Sadikov, Derek Suderman, Patricia Tai
Physicist: Varun-Singh Thakur

Saskatoon Cancer Centre
Site Investigator: Vijayananda Kundapur
Co-Investigators: Bryan Brunet, Ayesha Chandna, Naglaa Elsayed, Mohamed Emara, Suresh Kumar, Benjamin Maas, Syed Asim Noor, Vinita Sundaram, Haresh Vachhrajani, Moftah Younis
Physicist: Niranjan Venugopal

Hopital Charles LeMoyne
Site Investigator: Selma Mehiri
Co-Investigators: Jose Ayllon
Physicist: Vincent Cousineau-Daoust

© 2024 Swaminath A et al. *JAMA Oncology*.

eAppendix 2. Radiation Planning Guide

RANDOMIZED TRIAL OF STEREOTACTIC VERSUS CONVENTIONAL RADIOTHERAPY FOR STAGE I MEDICALLY INOPERABLE NON-SMALL CELL LUNG CANCER

LUSTRE

Radiation Planning Guide

for Stereotactic Body Radiotherapy (SBRT) and

Conventional Radiotherapy (Conventional RT) Techniques

Prepared by the

Radiotherapy Review Committee and the Ontario Clinical Oncology Group

At the Juravinski Hospital and Cancer Centre (JHCC)

ONTARIO CLINICAL ONCOLOGY GROUP

© 2024 Swaminath A et al. *JAMA Oncology*.

TABLE OF CONTENTS

1. PLANNING FOR EXPERIMENTAL (SBRT) TECHNIQUE 3

1.1. POSITIONING 3
1.1.1. Management of Internal Organ Motion 3
1.2. TREATMENT PLANNING 3
1.2.1. Image Acquisition 3
1.2.2. Target Volume Definitions 4
1.2.3. Organs at Risk (OAR) Definitions 4
1.3. DOSIMETRY 7
1.3.1. Beam Energy 7
1.3.2. Treatment Geometry 7
1.3.3. Prescription Dose and Coverage 7
1.3.4. Conformity and Low Dose Spillage 8
1.3.5. Dose Limits for OARs 9
1.4. TREATMENT VERIFICATION 10

2. PLANNING FOR CONTROL TECHNIQUE (CONVENTIONAL RT) 11

2.1. POSITIONING 11
2.1.1. Management of Internal Organ Motion 11
2.2. TREATMENT PLANNING 12
2.2.1. Image Acquisition 12
2.2.2. Target Volume Definitions 12
2.2.3. Organs at Risk (OAR) Definitions 12
2.3. DOSIMETRY 13
2.3.1. Beam Energy 13
2.3.2. Treatment Geometry 13
2.3.3. Prescription Dose and Coverage 13
2.3.4. Dose Limits for OARs 13
2.4. TREATMENT VERIFICATION 14

© 2024 Swaminath A et al. *JAMA Oncology*.

1. *Planning for Experimental (SBRT) Technique*

1.1. Positioning

Patients will be treated in the supine position with stable positioning to minimize the risk of intrafraction movement. A variety of immobilization systems can be used, including, but not limited to, a standard chest board and stereotactic frame conforming to the patients' external contours.

1.1.1. Management of Internal Organ Motion

Strategies to cope with respiratory motion are recommended in cases where significant motion can impair target localization and reproducibility. It is recommended that evaluation of tumour motion occur at the time of simulation, and if motion exceeds a threshold of 1 cm, then such methods should be employed. Approved methods include abdominal compression devices, tumour tracking (with fiduciary marker placement), respiratory gating (beam is on during a pre-specified point in the breathing cycle), or active breath-holding techniques. The final plan should indicate which method was used, if applicable.

1.2. Treatment Planning

1.2.1. Image Acquisition

A computed tomography (CT) scan is required as the primary dataset for contouring volumes and plan generation. Intravenous (IV) contrast is not mandatory, but can be used in cases of centrally located tumours, or in tumours causing local collapse/atelectasis. A 4-dimensional (4D) CT scan is required in order to account for respiratory motion. 4D CT is performed by correlating respiratory movement using external markers to projections taken at different points in the respiratory cycle. The images are then "binned" based on the phase of the cycle, and reconstructed. Images acquired during maximum exhale, maximum inhale, and a maximum

© 2024 Swaminath A et al. *JAMA Oncology*.

intensity projection (MIP) scan will be used to identify the targets. An average 4D CT scan may also be used to aid in target delineation or for correlation with image-guided radiotherapy (IGRT) on treatment. 4D CT images shall be assessed for artifacts. If significant artifacts exist either because of shallow or irregular breathing, then the 4D CT will be repeated. Persistent significant artifacts would render the 4D CT unreliable, and SBRT planning on study shall be abandoned. The primary dataset is based on institutional preference, with suggested datasets including average 4D CT, exhale 4D CT, or free-breathing CT. The primary dataset shall contain image slices ≤ 3mm, and include the entire thorax/lungs, with inclusion of the upper abdominal organs for lower lobe lesions, to mid-neck for apical lesions, and sufficient margin for accurate dose computation.

1.2.2. Target Volume Definitions

The gross tumour volume (GTV) is defined as the visible tumour seen on the planning CT simulation. No clinical target volume (CTV) delineation for microscopic disease will be performed. Recommended window/level settings for GTV definition are: -300 HU (level), and 1600 HU (width). Soft tissue/mediastinal windows may be used to identify chest wall, atelectasis, etc, but should not be used for GTV delineation. The internal target volume (ITV) accounts for changes in tumour position due to respiration (i.e. using the 4D CT) and is not required when breath-holding, gating, or tumour tracking techniques are used (i.e. the ITV is the same as the GTV). The planning target volume (PTV) will be an isotropic expansion of 0.5 cm around the ITV.

1.2.3. Organs at Risk (OAR) Definitions

The following nomenclature and standard contouring for OARs will be used.

© 2024 Swaminath A et al. *JAMA Oncology*.

SPINALCANAL – the bony boundary of the spinal cord. Include the relevant spinal canal that is within 10 cm superiorly and inferiorly to the PTV.

ESOPHAGUS – include both the muscular wall and lumen of the esophagus using mediastinal windows. Include the entire esophagus from below the cricoid cartilages to the gastro-esophageal junction.

RPLEXUS/LPLEXUS – the brachial plexus (right or left) shall be contoured in all cases when the tumour is located in the upper lobes. In lower lobe tumours this is not a required contour, but can be delineated in order to gain experience and comfort in contouring. Mediastinal or head & neck windows can be used to delineate this structure. The brachial plexus is defined by the subclavian vessels from their origin at the brachiocephalic or carotid bifurcations, and traversing along the path of the subclavian vein until it joins the axillary vein at the level of the 2nd rib. The entire neurovascular bundle should be defined in this manner, including the vein, artery, and nerve.

HEART – including the pericardial sac shall be contoured, on mediastinal windows. Delineation of the heart begins at the base of the great vessels, including both ventricles and atria, to its apex.

VESSELS – these include the superior vena cava, inferior vena cava, ascending aorta, aortic arch, and descending thoracic aorta. These vessels shall be contoured using mediastinal windows. The entire tunica and muscular layers will be included. For right-sided tumours, the

© 2024 Swaminath A et al. *JAMA Oncology*.

ascending/descending aorta can be omitted, for left-sided tumours the superior/inferior vena cava can be omitted. Aortic arch must be included regardless of the tumour location.

TRACHEA – includes all cartilaginous rings of the trachea as defined on lung windows, from its origin at the base of the cricoid cartilage, to 2 cm above the carina.

PROXTREE - The proximal bronchial tree is defined as the distal 2 cm of trachea, both right and left mainstem bronchi, the right and left upper lobe bronchi, bronchus intermedius, left lingular bronchus, right middle and lower lobe bronchus, and left lower lobe bronchus. It will be terminated at the bifurcation to the segmental bronchus.

SKIN – defined as the outer 0.5 cm of the body surface. It will include all skin surface on the planning CT, with the exception of the cranial and caudal limits.

RIBS – defined as the relevant rib structure within 5 cm of the PTV. Only the ribs will be contoured, not the intervening musculature.

STOMACH – for left lower lobe tumours, the entire stomach including the muscular and adventitial layers shall be included. This is not necessary for upper lobe tumours.

LIVER – the entire liver organ, excluding the porta hepatis and gallbladder will be contoured in cases of right lower lobe tumours.

BLUNG – both lungs (excluding the ITV) shall be contoured as per standard contouring methods.

LLUNG/RLUNG – left and right lungs will be labeled as separate contours (excluding the ITV).

© 2024 Swaminath A et al. *JAMA Oncology*.

1.3. Dosimetry

1.3.1. Beam Energy

A linear accelerator capable of 4-10 MV energy, equipped with a multi-leaf collimator of at most 10 mm “in-field” leaf width shall be used. *CyberKnife* (Accuray Inc., Sunnyvale, CA), a robotic linear accelerator of 6 MV, is permitted.

1.3.2. Treatment Geometry

Static 3D conformal SBRT may be used to achieve adequate dose distribution. Intensity modulated radiotherapy (IMRT) or volumetric modulated arc therapy (VMAT) techniques may be used in order to improve on conformality if necessary. Robotic radiosurgery plans are also permitted. The minimum field size for any one beam using 3-D conformal techniques shall be no less than 3 cm, since the PTV is within each beam aperture. For IMRT, VMAT, and robotic radiosurgery techniques, a minimum field size that yields accurate dose computation as established at each institution shall be employed.

1.3.3. Prescription Dose and Coverage

The prescription dose is 48Gy in 4 fractions of 12Gy for peripheral tumours, with a minimum of 24 hours separating each fraction (i.e. every other day). For lesions located within 1 cm of the VESSELS contour or within 2 cm of the PROXTREE contour, a risk-adapted dose of 60Gy in 8 daily fractions of 7.5Gy will be prescribed. The SBRT planning goal is for at least 95% of the PTV to be covered by at least 100% of the prescription dose (V100% ≥ 95%), and at least 99% of the PTV to be covered by at least 90% of the prescription dose (V90% ≥ 99%). Plans optimized using these criteria and using radiosurgery techniques described above, should result in the prescription dose corresponding to the 60-90% isodose line relative to the maximum dose. Lung heterogeneity corrections are required in order to achieve a realistic dose distribution. Dose calculations must be performed using Monte Carlo, superposition convolution, or Varian Acuros

© 2024 Swaminath A et al. *JAMA Oncology*.

XB algorithms. The maximum dose shall be within the PTV (ideally within the ITV), and not within an OAR.

1.3.4. Conformity and Low Dose Spillage

Conformality of the high dose volume is defined by the ratio of the prescription (48/60 Gy) isodose volume and the PTV volume ($R_{100\%}$). Intermediate/low dose spillage will be quantified using the $R_{50\%}$, the ratio between the 50% prescription isodose volume (24/30 Gy) and the PTV volume. Furthermore, the dose at 2 cm from the PTV (D_{2cm}) will be evaluated. The table below (modified from Hurkmans *et al.*, 2010) outlines the protocol requirements for conformity and low dose spillage.

Table 1: Dose conformity requirements and definition of protocol deviations. $R_{100\%}$ and $R_{50\%}$ are the ratios of the 100 and 50% prescription isodose volumes to the PTV, respectively. $D_{2\ cm}$ is the maximum dose 2 cm away from the PTV as a percentage of the prescribed dose.

PTV (cm^3)	$R_{100\%}$		$R_{50\%}$		$D_{2\ cm}$ (%)	
	Deviation		Deviation		Deviation	
	None	Minor	None	Minor	None	Minor
0-20	<1.25	1.25-1.40	<12	12-14	<65	65-75
20-40	<1.15	1.15-1.25	<9	9-11	<70	70-80
>40	<1.10	1.10-1.20	<6	6-8	<70	70-80

© 2024 Swaminath A et al. *JAMA Oncology*.

1.3.5. Dose Limits for OARs

Normal tissue restrictions are listed in the table below. In cases of overlap between PTV and OARs, dose constraints on OARs will be considered higher priority (with the exception of rib dose). Not meeting dose-volume histogram (DVH) criteria for OARs will be considered a minor violation and the case should be reviewed by the study chair for the degree of overlap prior to continuation on study.

Table 2: DVH Limits for the SBRT arm (48 Gy in 4 fractions):

Organ	Maximum Point Dose (dose per fraction) [Gy]	Critical Volume [cm^3]	Critical Volume Dose (dose per fraction) [Gy]
SPINALCANAL	27 (6.75)	1	18 (4.5)
ESOPHAGUS	30 (7.5)	5	19 (4.75)
R/LPLEXUS (if applicable)	27 (6.75)		
HEART	35 (8.75)	15	29 (7.25)
VESSELS	48 (12)	10	40 (10)
TRACHEA/PROXTREE	40 (10)	5	32 (8)
SKIN	36 (9)	10	33 (8.25)
RIBS	50 (12.5)	5	40 (10)
STOMACH (if applicable)	28 (7)	1	21 (5.25)
BLUNG		1000	13 (3.25)
		**Critical Volume [%]**	**Critical Volume Dose (dose per fraction) [Gy]**
BLUNG		10	20 (5)

Table 3: DVH Limits for the SBRT arm (60 Gy in 8 fractions):

© 2024 Swaminath A et al. *JAMA Oncology*.

Organ	Maximum Point Dose (dose per fraction) [Gy]	Critical Volume [cm^3]	Critical Volume Dose (dose per fraction) [Gy]
SPINALCANAL	32(4)	1	22(2.75)
ESOPHAGUS	40 (5)	5	22 (5)
R/LPLEXUS (if applicable)	38 (4.75)		
HEART	64 (8)	10	60 (7.5)
VESSELS	64 (8)	10	60 (7.5)
TRACHEA/PROXTREE	64 (8)	5	60 (7.5)
SKIN	45 (5.6)	10	40 (5)
RIBS	60 (7.5)	5	50 (6.25)
STOMACH (if applicable)	40 (5)	1	36 (4.5)
BLUNG		1000	18 (2.25)
		**Critical Volume [%]**	**Critical Volume Dose (dose per fraction) [Gy]**
BLUNG		10	20 (2.5)

1.4. Treatment Verification

It is strongly advised that patients have a mock setup (particularly with dosimetry involving non-coplanar beam angles) prior to day 1 of treatment. This can be achieved by the patient having a day 0 setup on the machine, with or without the presence of the patient. If non-coplanar beams are being used, it is advised that the patient be present for the day 0 to ensure no gantry collisions occur.

Patients treated on linear accelerators will require daily kV cone-beam CT (CBCT) imaging with dedicated matching, first to bone, and then to soft tissue (ITV) prior to each fraction. The average position of the tumour based on CBCT should fall within the ITV and PTV. In cases of

© 2024 Swaminath A et al. *JAMA Oncology*.

close proximity to OARs, these contours should be propagated from the planning system to the CBCT console, along with the appropriate avoidance isodose line. *Recommended* tolerances for translational (x,y,z) and rotational (pitch, roll, yaw) are ≤ 2 mm and ≤ 3 degrees, respectively. Patient rotations exceeding 3 degrees require an adjustment in patient setup. For translations greater than 2 mm but less than 10 mm, a couch shift should be performed. If a manual couch shift is performed, then a verification CBCT is suggested. Shifts beyond 10 mm require re-imaging after the couch is translated to confirm positioning. If the residual shift following couch translation remains beyond 10 mm, a complete re-setup and re-imaging is required. Replanning is strongly recommended when large shifts (> 10 mm) are necessary on two occasions during the entire treatment course (all fractions). Patients treated with robotic radiosurgery will require tumour tracking with fiducial marker placement prior to treatment, and respiratory motion modeling using the Synchrony system (Accuray Inc., Sunnyvale, CA).

2. *Planning for Control Technique (Conventional RT)*

2.1. Positioning

Patients will be treated in the supine position with arms up. A chest board or vacuum lock device is adequate for immobilization; stereotactic frames are not required.

2.1.1. Management of Internal Organ Motion

Strategies to limit internal organ movement are not required. A 4D CT can be used in order to generate an ITV, but is not necessary.

© 2024 Swaminath A et al. *JAMA Oncology*.

2.2. Treatment Planning

2.2.1. Image Acquisition

A computed tomography (CT) scan is required as the primary dataset for contouring volumes and plan generation. Intravenous (IV) contrast is not mandatory, but can be used in cases of centrally located tumours, or in tumours causing local collapse/atelectasis. A 4-dimensional (4D) CT scan is not required, but can be used if centre policy. The primary dataset shall contain image slices at most 3 mm thick, and should include the entire thorax/lungs, with inclusion of the upper abdominal organs for lower lobe lesions, and to mid-neck for apical lesions. Additional margin in the superior-inferior direction must be added to ensure accurate dose computation.

2.2.2. Target Volume Definitions

The gross tumour volume (GTV) is defined as the visible tumour seen on the planning CT simulation. No clinical target volume (CTV) delineation for microscopic disease will be performed. Recommended window/level settings for GTV definition are: -300 HU (level), and 1600 HU (width). Soft tissue/mediastinal windows may be used to identify chest wall, atelectasis, etc, but should not be used for GTV delineation. The internal target volume (ITV) is defined using the 4D CT if available. The planning target volume (PTV) will be dependent on the extent of imaging modalities used. If a 4D CT is used to generate an ITV, and daily kV CBCT image guidance with bone and soft tissue matching is being employed, then a 0.5 cm isotropic PTV margin is acceptable. If no 4D CT is being used (no ITV expansion) OR image guidance consists of either daily kV portal images or kV CBCT with bone matching only, then PTV margin must be greater than 0.5 cm, with 1 cm isotropic recommended.

2.2.3. Organs at Risk (OAR) Definitions

The nomenclature and standard contouring for OARs are described in section 1.2.3.

© 2024 Swaminath A et al. *JAMA Oncology*.

2.3. Dosimetry

2.3.1. Beam Energy

A linear accelerator capable of 4-10 MV energy, equipped with a multi-leaf collimator of at most 10 mm “in-field” leaf width shall be used.

2.3.2. Treatment Geometry

No mediastinal/hilar nodal radiation is to be performed. CT planning is to be executed, with 3D conformal RT techniques. IMRT or VMAT is permitted (if centres are capable of planning using this technique). At least 3 beams using conformal techniques should be used to cover the PTV adequately.

2.3.3. Prescription Dose and Coverage

The prescription dose is 60Gy in 15 fractions, 4Gy per fraction delivered daily (Monday-Friday) for 3 weeks. Conventional RT planning goals are as follows: at least 95% of the PTV is to be covered by at least 95% of the prescription dose (V95%≥95%). Lung heterogeneity corrections must be performed using Monte Carlo, superposition convolution, or Varian Acuros XB algorithms to achieve a more realistic dose distribution. The maximum point dose must be within the PTV and not exceed 110% of the prescription dose.

2.3.4. Dose Limits for OARs

Normal tissue restrictions are listed in the table below. In cases of overlap between PTV and OARs, dose constraints on OARs will be considered higher priority (with the exception of rib dose).

Table 4: DVH Limits for the Conventional RT arm (60 Gy in 15 fractions):

© 2024 Swaminath A et al. *JAMA Oncology*.

Organ	Maximum Point Dose (dose per fraction) [Gy]	Critical Volume [%]	Critical Volume Dose (dose per fraction) [Gy]
SPINALCANAL	36 (2.27)	-	-
ESOPHAGUS	48 (3.2)	-	-
L/RPLEXUS (if applicable)	50 (3.33)	-	-
HEART	66 (4.4)	-	-
VESSELS	66 (4.4)	-	-
TRACHEA/PROXTREE	66 (4.4)	-	-
SKIN	45 (3)	-	-
RIBS	66 (4.4)	-	-
STOMACH (if applicable)	48 (3.2)	-	-
BLUNG		15	20
		30	15

2.4. Treatment Verification

Daily kV orthogonal projections or kV cone beam CT images will be taken for purposes of treatment verification. Image matching to bone using daily kV portal images or kV CBCT should be performed. According to institutional policies, if daily CBCT includes soft-tissue matching (to ITV generated from 4D CT), then PTV margins can be smaller (see section 2.2.2). Tolerances re per institutional standard.

© 2024 Swaminath A et al. *JAMA Oncology*.

eAppendix 3. Definition of Outcomes and Censoring Rules

Local control (LC) was defined using the following criteria: (a) primary tumor failure (enlargement of the lesion longest diameter of at least 20% from the smallest size on 2 or more serial CT examinations at least 3 months apart, with an absolute increase of at least 5 mm) or (b) marginal failure (appearance of a new lesion within 1 cm of the target volume with similar growth criteria). In cases suspicious of local recurrence, histologic evidence was recommended. Where a biopsy was not possible or deemed to be high risk, an FDG-PET was obtained to assess response. A standardized uptake value (SUV) > 5, or an increase in metabolic uptake of 25% compared with initial tumor SUV, was deemed to be recurrence.

For the LC analysis, local failure was defined as time from randomization to primary tumor or marginal failure at any timepoint with censoring only at last follow-up or death. Patients a with local failure either before or after a regional or distant failure were included.

Secondary outcomes included event-free survival (EFS - either 1st local/regional/distant lung cancer progression, new primary cancer, or death from any cause), and overall survival (OS - death from any cause).

For EFS and OS, subjects were censored if event-free at date of last follow-up.

© 2024 Swaminath A et al. *JAMA Oncology*.

eAppendix 4. Baseline Characteristics

**Characteristic**	**SBRT** n = 154	**CRT** n = 79
Age (years): *mean (SD)*	76 (8)	75 (8)
Charlson Score: *mean (SD)*	1.9 (1.6)	1.9 (1.6)
Primary Tumour Diameter: *mean (SD)*	2.5 (1.0)	2.4 (1.0)
Sex: *n (%)*		
Male	80 (52)	39 (50)
Female	74 (48)	40 (50)
Location: *n (%)*		
Central	45 (29)	19 (24)
Peripheral	109 (71)	60 (76)
Stage: *n (%)*		
T1	110 (71)	56 (71)
T2	44 (29)	23 (29)
Confirmation: *n (%)*		
Histological	81 (53)	34 (43)
Imaging	73 (47)	45 (57)
Pathological Subtype: *n*		
Squamous Cell	30	14
Adenocarcinoma	47	18
Large cell carcinoma	1	1
Other / Not Specified	3	1

*SBRT – Stereotactic Body Radiotherapy; CRT – Conventionally Hypofractionated Radiotherapy

© 2024 Swaminath A et al. *JAMA Oncology*.

eAppendix 5. Cumulative Incidence of Local Failure, Patterns of Failure, and Causes of Death

Cumulative Incidence of Local Failure

The probability of local recurrence at any time was estimated with the use of the cumulative incidence function, accounting for death as a competing event, and the corresponding 95% confidence intervals were estimated with the use of delta methods. For the competing risk analysis, the sub-distribution HR was determined using the Fine-Gray model.

3-year cumulative incidence of local failure for SBRT = 10.7% (95% CI: 6.4, 16.3) and

3-year cumulative incidence of local failure for CRT = 16.7% (95% CI: 9.4, 25.8)

Sub-distribution hazard ratio (HR) = 0.59 with 95% CI: 0.30, 1.14. p=0.12

Patterns of 1st Failure for EFS

Pattern	CRT (n=79)	SBRT (n=154)
Local Recurrence	12 (15.2%)	16 (10.4%)
Regional Recurrence	6 (7.6%)	13 (8.4%)
Distant Recurrence	5 (6.3%)	8 (5.2%)
Death	15 (19.0%)	38 (24.7%)
New Primary Cancer	11 (13.9%)	16 (10.4%)

Causes of Death

Cause	CRT (n=79)	SBRT (n=154)
Lung Cancer	22 (27.8%)	30 (19.5%)
Co-Morbidity (eg cardiac or respiratory disease)	11 (13.9%)	20 (13.0%)
Radiation Related	0 (0.0%)	1 (0.6%)
Second Cancer	1 (1.3%)	2 (1.3%)
Unknown	4 (5.1%)	20 (13.0%)
TOTAL	38 (48.1%)	73 (47.4%)

© 2024 Swaminath A et al. *JAMA Oncology*.

eAppendix 6. Further Toxic Effects Details

Grade 3, 4, 5 Late Toxicity Events

**Grade 3, 4 or 5 All Patients ***	**SBRT** n = 154	**CRT** n = 79
Long-term Toxicity (> 3 – 36 months): *n (%)*		
Fatigue	1 (<1)	1 (1)
Dyspnea	3 (2)	2 (3)
Chest wall pain	2 (1)	1 (1)
Pneumonitis	0 (0)	1 (1)
Infections, Pneumonia	1 (<1)	0 (0)
Bronchial Stricture	1 (<1)	0 (0)
Bronchial Obstruction	1 (<1)	0 (0)
Atelectasis*	1 (<1)	0 (0)
Hemoptysis	1 (<1)	0 (0)
**Total**	**11 (7)**	**5 (6)**

**Grade 3, 4 or 5 Central Tumors Only**	**SBRT** n = 45	**CRT** n = 19
Long-term Toxicity (> 3 – 36 months): *n (%)*		
Fatigue	1 (<1)	0 (0)
Dyspnea	2 (2)	1 (1)
Chest wall pain	1 (<1)	0 (0)
Pneumonitis	0 (0)	1 (1)
Bronchial Stricture	1 (<1)	0 (0)
Bronchial Obstruction*	1 (<1)	0 (0)
Atelectasis*	1 (<1)	0 (0)
Hemoptysis	1 (<1)	0 (0)
**Total**	**8 (18)**	**2 (11)**

SBRT – Stereotactic Body Radiotherapy; CRT – Conventionally Hypofractionated Radiotherapy; *Reported toxicities are by event, multiple toxicities may have occurred in the same patient

© 2024 Swaminath A et al. *JAMA Oncology*.

eAppendix 7. SBRT vs CRT RCT Dose Comparison

TRIAL	SBRT ABSOLUTE DOSE	CRT ABSOLUTE DOSE	BED10 SBRT	BED10 CRT	EQD2 SBRT	EQD2 CRT
LUSTRE	48 Gy/4 fractions<br>60 Gy/8 fractions	60 Gy/15 fractions	105.6 Gy<br>105.0 Gy	84.0 Gy	88.0 Gy<br>87.5 Gy	70.0 Gy
SPACE	45 Gy/3 fractions	70 Gy/35 fractions	112.5 Gy	84.0 Gy	93.8 Gy	70.0 Gy
CHISEL	54 Gy/3 fractions<br>48 Gy/4 fractions	66 Gy/33 fractions<br>50 Gy/20 fractions	151.2 Gy<br>105.6 Gy	79.2 Gy<br>62.5 Gy	126.0 Gy<br>88.0 Gy	66.0 Gy<br>52.1 Gy

BED – biologically equivalent dose (using alpha/beta ratio of 10 for lung cancer)
EQD2 – equivalent dose in 2 Gy/day (using alpha/beta ratio of 10 for lung cancer)

© 2024 Swaminath A et al. *JAMA Oncology*.

eAppendix 8. Recruitment Table by Center

**Clinical Center**	**Recruitment**
Juravinski Cancer Centre	21
Cancer Centre of Southeastern Ontario at Kingston	12
Niagara Health System, St. Catharines	3
London Regional Cancer Centre	2
Thunder Bay Regional Health Sciences Centre	10
Windsor Regional Cancer Centre	16
BC Cancer - Vancouver Island Centre	0
CancerCare Manitoba	17
Tom Baker Cancer Centre	5
Cross Cancer Institute	2
Horizon Health Network – Saint John Regional Hospital	3
McGill University Health Centre (MUHC) - Glen site	22
Hopital Maisonneuve - Rosemont	17
CHUM - Centre Hospitalier de L'Université de Montréal	6
Allan Blair Cancer Centre	11
Saskatoon Cancer Centre	84
Hopital Charles LeMoyne	2
**Total:**	**233**

© 2024 Swaminath A et al. *JAMA Oncology*.
