# Supplementary material for: Stereotactic vs Hypofractionated Radiotherapy for Inoperable Stage I Non–Small Cell Lung Cancer: The LUSTRE Phase 3 Randomized Clinical Trial
Source: JAMA Oncol. 2024 Sep 19;10(11):1571–5. doi: 10.1001/jamaoncol.2024.3089 (PMC11413752; doi:10.1001/jamaoncol.2024.3089)
Supplement: Supplement 3. — Data Sharing Statement [file jamaoncol-e243089-s003.pdf]

Data Sharing Statement

Swaminath. Stereotactic vs Hypofractionated Radiotherapy for Inoperable Stage I Non–Small Cell Lung Cancer. *JAMA Oncol*. Published September 19, 2024.
doi:10.1001/jamaoncol.2024.3089

Data
Data available: No
